# ZnO nucleation into trititanate nanotubes by ALD equipment techniques, a new way to functionalize layered metal oxides

**DOI:** 10.1038/s41598-021-86722-0

**Published:** 2021-04-08

**Authors:** Mabel Moreno, Miryam Arredondo, Quentin M. Ramasse, Matthew McLaren, Philine Stötzner, Stefan Förster, Eglantina Benavente, Caterina Salgado, Sindy Devis, Paula Solar, Luis Velasquez, Guillermo González

**Affiliations:** 1 Universidad SEK, Instituto de investigación Interdisciplinar en Ciencias Biomédicas SEK (I3CBSEK), Facultad Ciencias de la Salud, Fernando Manterola 0789, Providencia, Santiago Chile; 2 Max Planck Institute of Microstructure Physics, Weinberg 2, 06120 Halle, Germany; 3 Facultad de Ciencias, Universidad de Chile, Las Palmeras 3425, Nuñoa, Santiago Chile; 4 Queen’s University Belfast, University Rd, Belfast, BT7 1NN UK; 5 SuperSTEM Laboratory, STFC Daresbury Campus, Daresbury, WA4 4AD UK; 6 School of Chemical and Process Engineering, University of Leeds, Leeds, LS2 9JT UK; 7 Living Systems Institute, University of Exeter, Exeter, EX4 4QD UK; 8 Martin-Luther-Universität Halle-Wittenberg, Halle, Germany; 9 Departamento de Química, Facultad de Ciencias Naturales, Matemática y Medio Ambiente, Universidad Tecnológica Metropolitana, Santiago, Chile; 10 Programa Institucional de Fomento a la Investigación, Desarrollo e Innovación (PIDi), Universidad Tecnológica Metropolitana, Santiago, Chile

**Keywords:** Nanoparticle synthesis, Structural properties

## Abstract

In this contribution, we explore the potential of atomic layer deposition (ALD) techniques for developing new semiconductor metal oxide composites. Specifically, we investigate the functionalization of multi-wall trititanate nanotubes, H_2_Ti_3_O_7_ NTs (sample T1) with zinc oxide employing two different ALD approaches: vapor phase metalation (VPM) using diethylzinc (Zn(C_2_H_5_)_2_, DEZ) as a unique ALD precursor, and multiple pulsed vapor phase infiltration (MPI) using DEZ and water as precursors. We obtained two different types of tubular H_2_Ti_3_O_7_ species containing ZnO in their structures. Multi-wall trititanate nanotubes with ZnO intercalated inside the tube wall sheets were the main products from the VPM infiltration (sample T2). On the other hand, MPI (sample T3) principally leads to single-wall nanotubes with a ZnO hierarchical bi-modal functionalization, thin film coating, and surface decorated with ZnO particles. The products were mainly characterized by electron microscopy, energy dispersive X-ray, powder X-ray diffraction, Fourier transform infrared spectroscopy, and X-ray photoelectron spectroscopy. An initial evaluation of the optical characteristics of the products demonstrated that they behaved as semiconductors. The IR study revealed the role of water, endogenous and/or exogenous, in determining the structure and properties of the products. The results confirm that ALD is a versatile tool, promising for developing tailor-made semiconductor materials.

## Introduction

Atomic layer deposition (ALD), like other vapour deposition techniques^1^, has proven to be a suitable tool for solvent-free fabrication of advanced metal oxide-based functional materials potentially useful to address pressing global problems such as the increase in environmental pollution, the lack of clean energy sources or the development of new healthcare technologies^2,3^. Since solar energy addresses several of these concerns, ALD strategies to improve both the absorption and the efficiency of broadband semiconductor-based photocatalysts such as ZnO or TiO_2_ under visible light are receiving increasing attention^4,5^.

Zinc oxide is a semiconductor, which, beyond having physicochemical environment-friendly properties similar to those of TiO_2_, possesses interesting photophysical properties such as high exciton binding energy and quite large electron mobility. It has thus been studied extensively in recent years as an alternative to TiO_2_^6,7^. ZnO's outstanding electron mobility and optical properties make it interesting as a material for electro-optical applications, for example, for liquid crystal displays^8^, light-emitting diodes^9^, or thin-film transistors^10^. ZnO deposition by ALD has been known since the dawn of this technique^11^. Among a variety of precursors, diethyl zinc (DEZ) is the most popular due to its high volatility and great reactivity, even at room temperature^12^. Recently, Cai et al. published an extensive study on the growth of ZnO in different substrates in a wide temperature range (30–250 °C) concluding that the highest growth rate is reached at 150 °C, the deposited material is always wurtzite with a high degree of crystallinity, and each DEZ molecule generally reacts with 1.5 –OH surface sites^13^.

The low quantum yield of broadband semiconductors such as pure TiO_2_ and ZnO, as well as the photodegradation of ZnO, make their use difficult in photocatalytic applications^14^. Suitable semiconductor heterojunctions between two or more semiconductors in which synergistic interactions between the components promote separation and transfer of photogenerated charges and reduce recombination rates have been shown to significantly improve photocatalytic processes^15^. Even mechanical mixing of ZnO/TiO_2_ with 1–7% TiO_2_ catalyses the photodegradation of organic dyes more efficiently than the semiconductors on their own, which has been attributed to the formation of p-ZnO by substitution of Ti^4+^ by Zn^2+^ in the interface between both oxides^16^. Recently, more intimate mixtures of ZnO wurtzite and TiO_2_ anatase/rutile (Degussa P25) prepared by hydrothermal treatment in an alkaline medium have been reported, resulting in nanostructures with optimal physicochemical and photocatalytic properties when the ZnO:TiO_2_ ratio is 1:1^17^. Also working under hydrothermal conditions but from the oxide precursors (zinc salt, and titanium (IV) isopropoxide), the preparation of nanoflakes decorated with TiO_2_ nanoparticles capable of catalysing the degradation of dyes under sunlight quickly has been reported^18^. The great ability of ZnO to produce various morphologies and specific nanostructures facilitates the rational design and production of functional hetero photocatalysts by coupling with semiconductors with complementary properties^19^. Hybrid nanorod (NR) of wurtzite ZnO covered at the tips with amorphous anatase/rutile TiO_2_ nanoparticles fabricated by hydrothermal treatment (180 °C, 24 h) of previously prepared nanorods and TiO_2_ nanoparticles, showed photodegradation of methylene blue some five times faster than the best of its components (ZnO NR)^20^.

Semiconductor coupling is often more efficient when the composite material has a core–shell configuration where one of the components (core) is completely covered by the second (shell)^21^. The activity of the photocatalysts obtained by controlled deposition of a TiO_2_ shell on structures similar to ZnO flowers, proven by the photodegradation of methylene blue, increases with the increase of the TiO_2_ coverage, reaching higher yields than those of its precursors, but only up to a precise shell thickness threshold^22^. ALD techniques are particularly suitable for producing ZnO-based core–shell nanostructures using homogeneous and conformal coatings with precisely controlled thicknesses. Such a strategy has proved to be useful for investigating the processes, which determine the behaviour of semiconductor heterojunctions, as well as to develop energy conversion devices. Among them, solar cells^23^, photoelectrochemical water splitting systems^24^, electrode materials for lithium batteries^25^, or photovoltaic cells^26^. ALD has proven to be a unique technique for realizing the superior photocatalytic activity expected from the photophysical properties of ZnO by precisely fitting around it a TiO_2_ layer that is thick enough to protect ZnO from photocorrosion but also sufficiently thin to avoid recombination of charge carriers within the TiO_2_ layer^27,28^. Combining electrospinning techniques Kayaci et al.^29^, attained to encapsulated core nanowires of TiO_2_ and ZnO into ~ 10 nm-thick shells of ZnO (TiO_2_–ZnO) and TiO_2_ (ZnO–TiO_2_), respectively, leading to materials able to react with the environment selectively through photo-generated holes or electrons. Protection of the ZnO surface with a titania ALD-layer proved to be useful to improve the efficiency of dye-sensitized solar cells, which allowed to produce chemically stable ZnO-based photo anodes, much faster than TiO_2_ for transport of injected electrons^30^.

ALD achieves film growth through the chemical reaction of two typically gaseous reactants (precursors) on a surface (substrate), where the precursors are supplied, one at a time, in a series of sequential non-overlapping pulses by a procedure guaranteeing that no more than one of the precursors is present in the reactor at any time. Thus, each precursor of ALD interacts with the substrate in a process only limited by the reactive sites available on the surface, leading to a self-limited event^12,31–33,34^ and the deposition of one monolayer, i.e. ~ 0.1 nm growth per cycle (GPC). The first TiO_2_ anatase and ZnO monolayers were reported by Kol'tsov, S. I. in 1970^35^, utilizing TiCl_4_ and H_2_O as precursors on Si substrates at 180 °C and, by Stepanova S.I. et al., in 1977, by exposing the precursor ZnCl_2_ on silica gel at 450–600 °C followed by hydrolysation at 180 °C, respectively^35^. Since then, numerous studies have been published on the conformal growth of ALD of TiO_2_ and ZnO films^13,36^, demonstrating the ability of this technique to produce uniform flat surfaces without pores on a large scale^13,37,38^, as well as the formation of templated nanostructures with aspect ratios as large as 10E^5^ ^39^. The use of low working temperatures (less than 200 °C) also allows the use of natural or synthetic organic templates^40^, including 3D polymer nano-networks^41^.

**Figure 1:**
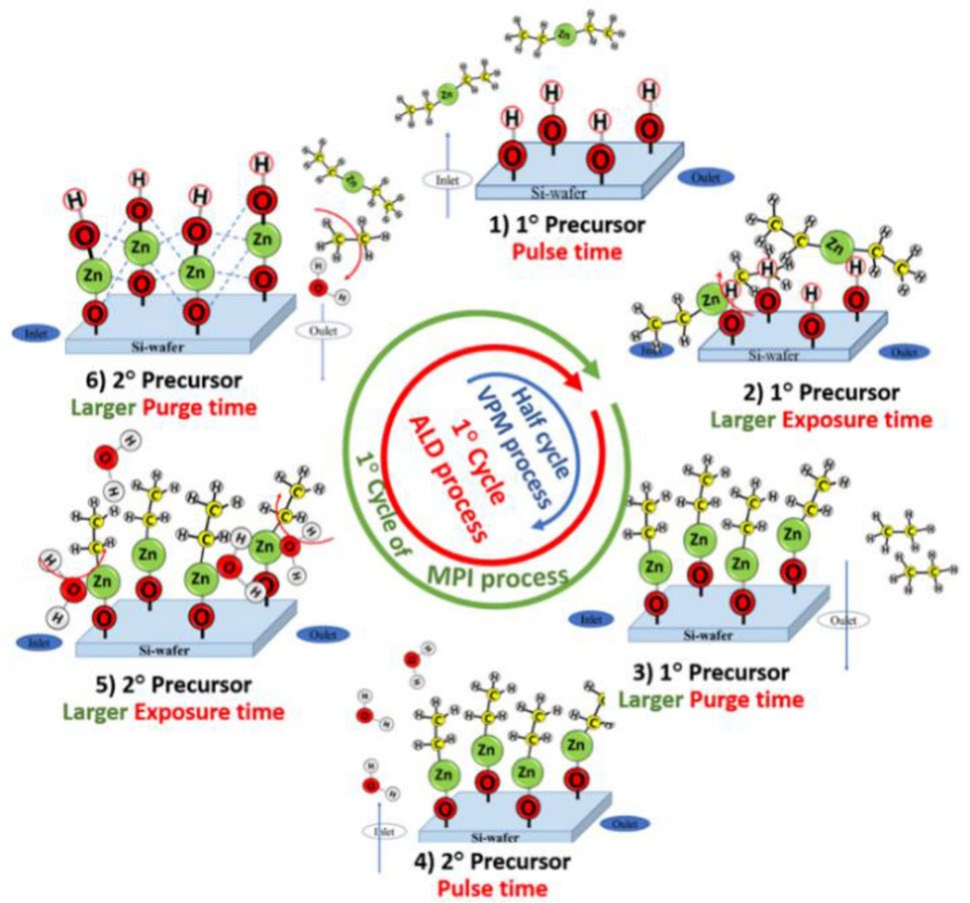
Scheme for the synthesis of zinc oxide by atomic layer deposition (ALD). (1) Diethylzinc (DEZ) pulse injection, (2) chemisorption of DEZ upon the Si wafer surface, (3) purge of unreacted DEZ and by-products, (4) H_2_O pulse injection, (5) reaction of water with absorbed DEZ, and (6) purge of excess H_2_O and by-products.

The intrinsic versatility of ALD is further enhanced by a series of methodologies derived from the same principles: Molecular layer deposition (MLD)^42^, in which at least one of the precursors is an organic molecule; MPI^43–48^, which uses longer exposures of the substrate to both types of ALD precursors to favour metal infiltration; and VPM^49,50^, which exposes the substrate solely to the metal precursor (half cycle, see Fig. 1), but for longer time periods. The first report of the MPI technique was provided by the pioneering contribution of George’s group^51^, who introduced the deposition of Al_2_O_3_ by sequential TMA/H_2_O ALD cycles into polymers (e.g., polystyrene) using long exposure times; thereafter the functionalization of polymers by metal infiltration has received much attention^43,45–47,51,52^. From a biological application point of view, the mechanical properties improvement of a biomaterial achieved by MPI metal infiltration into the proteins of spider dragline silks using organo-metallic ALD precursors of Zn, Ti, or Al^44^, is particularly interesting. Through the same procedure, it has been possible to infiltrate Zn, Al, or Ti oxide into a variety of materials like cellulose^50^, polyamide-6^45^, polyester fibers^46^, polyimide^47^, conductive polymers^51^, or carbonaceous materials^53^. Meanwhile through VPM or other similar one-precursor infiltration method, where reaction with the organometallic is possible only at determined substrate nucleophilic centers, highly specific metalation has been achieved. This is observed for instance in the metalation of a pre-prepared Zirconium(IV) MOF (metal–organic framework) using the ALD precursors trimethylaluminium or diethylzinc^48^, or in the selective metalation of the methylmethacrylate (PMMA) blocks in its block-copolymer with phenylstyrene (PMMA-b-PS)^54^. In this way, the MPI and VPM processes have proved to be successful tools not only in the synthesis of metalized complexes but also in the creation of soft organic/inorganic hybrid materials with synergistically enhanced properties.

The decoration of an anodized TiO_2_ array with ZnO nanoparticles has also been performed by sol–gel^55^, thermal decomposition^37^, electrodeposition^38^, or ALD methods^56^. All these titania nanotubes rely on TiO_2_–anatase nanostructures where the growth of ZnO ALD is mainly induced by the surface population of OH groups. There are also the so-called hydrothermal TiO_2_ nanotubes (HTNT) prepared for the first time by Kasuga et al. in the late 1990s, that are structurally and morphologically different from anatase nanotubes^57^. HTNTs are multi-walled and open-ended tubular structures formed by winding sheets of hydrogen titanate H_2_Ti_3_O_7_^58^ whose Ti–O lattice is more like metastable TiO_2_ (B) than anatase^59^. The laminar structure together with the richness of nucleophilic centers distributed hierarchically on the surface, internal cavity and interlaminar spaces within the walls of the HTNT makes them a complex but interesting scaffold for the nucleation of ZnO until now unexplored.

In this paper, we explore the potential of ALD techniques for the development of new tailor-made semiconductor metal oxide composites based on ZnO and H_2_Ti_3_O_7_ nanotubes. Specifically, we investigate the deposition of ZnO on multi-wall H_2_Ti_3_O_7_ nanotubes (sample T1) using two different ALD approaches: VPM using diethylzinc (Zn(C_2_H_5_)_2_, DEZ) as a unique ALD precursor (sample T2) and MPI of H_2_Ti_3_O_7_ using DEZ and water as ALD precursors (sample T3).

## Results and discussion

### Characterization of H_2_Ti_2_O_7_ NTs

**Figure 2:**
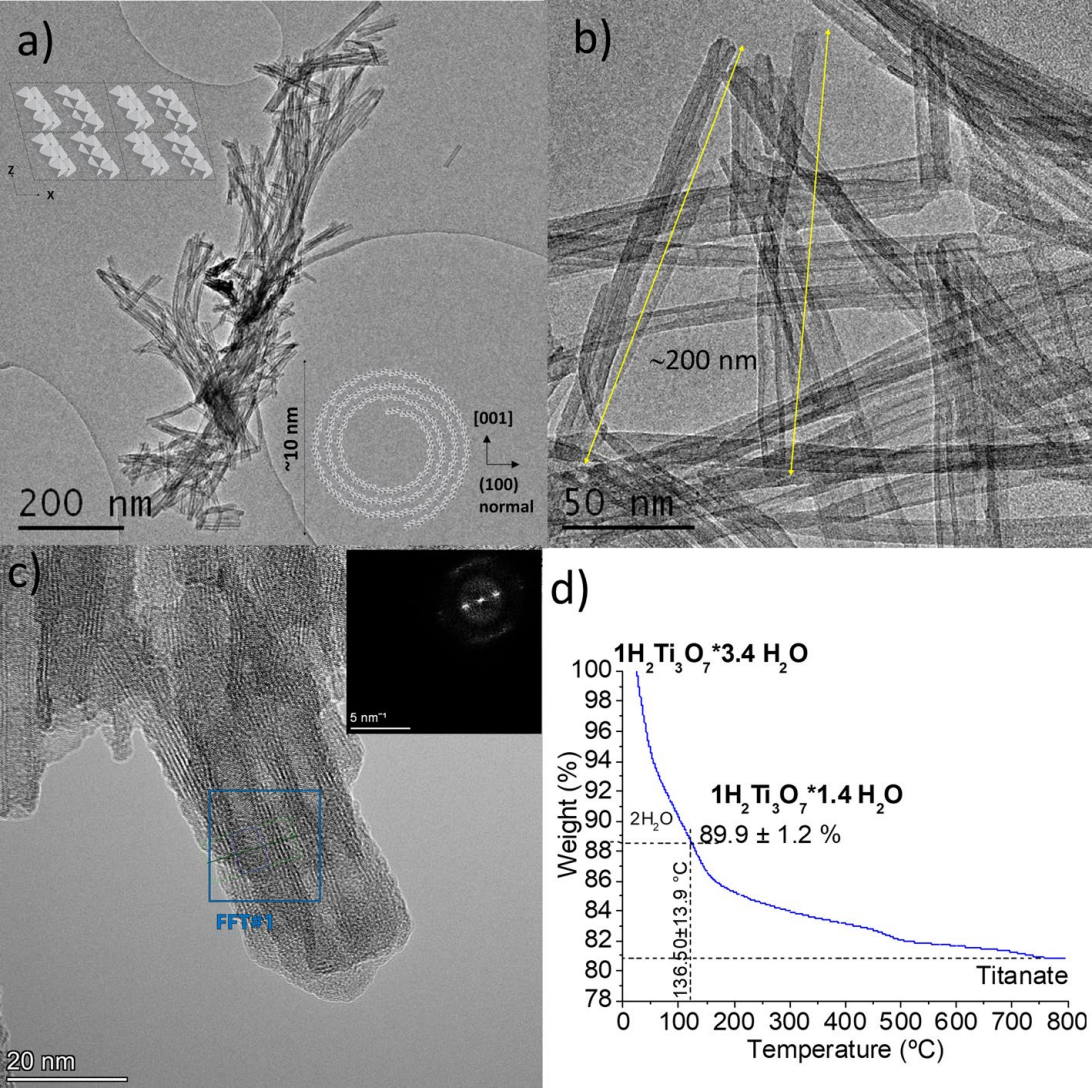
T1 TEM micrographs of sample T1 at different magnifications (**a**, **b**) in a JEOL JEM-2011 operated at 200 kV, schematic drawing of the structure of H_2_Ti_3_O_7_ NTs (inset in **a**), and (**c**) in a TALOS G-2 TEM at 200 kV. The inset is a Fourier transform taken from the area of the tube only marked by the blue box. A more detailed analysis of the TEM micrographs (Figure S2 in ESI) showed tubular cavities with diameters in the range of 9–11 nm as well as difference in thicknesses of opposite walls of the tubes). (**d**) Thermogravimetric analysis of T1.

The transmission electron microscopy (TEM) micrograph in Fig. 2a shows that T1 is composed of fiber-shaped particles with very large aspect ratios, while Fig. 2b,c shows some higher magnification transmission electron microscopy (TEM) micrographs typical of the nanotubes used for this study. The sample was entirely composed of multi-wall, open-ended nanotubes with lengths and outer diameters of ca 200 nm and 10.4 ± 1.8 nm on average, respectively. The tubes walls with thicknesses of about 3.3 nm generally consisted of 3–6 concentric sheets defining interlaminar spaces of about 0.80 ± 0.07 nm (Table 1), in line with earlier reports^58,60^. Figure 2c shows a set of side-by-side tubes with walls (A) ~ 5 nm wide, composed of 6 layers and interlamellar spaces (B) of ~ 0.70 nm on average. The Fourier transform (FFT) from the tube-wall (shown in the inset) confirms its laminar structure and the already mentioned spacings. The composition of the compound was deduced from elemental thermogravimetric (TG) and energy dispersive X-ray (EDX) analysis (see Fig. 2d and Figure S1 in electronic supplementary information (ESI)) of the product corresponds approximately to a stoichiometry of H_2_Ti_3_O_7_·3.14H_2_O, contaminated by a small amount of C and N, probably arising from the surfactant, and by un-exchanged Na ions.

**Table 1:** Dimensions of samples T1, T2, and T3 in nm. Total diameter (T.d), inner diameter (I.d), layers (L), and layer thickness (L.T).

Sample	T.d in nm	I.d in nm	W in nm	L in nm	L.T in nm
T1^a^	10.4 ± 1.8	3.9 ± 0.5	3.2 ± 0.9	4.0 ± 1.1	0.80 ± 0.07
T2^b^	12.6 ± 2.4	3.0 ± 1.3	4.7 ± 1.6	3.5 ± 1.3	1.45 ± 0.48
T3^c^	13.4 ± 2.9	2.9 ± 0.8	5.3 ± 2.0	3.0 ± 1.2	2.05 ± 0.85

^a–c^Amount of nanostructures tested: a. 9 NTs; b. 67 NTs; c.43 NTs.

**Figure 3:**
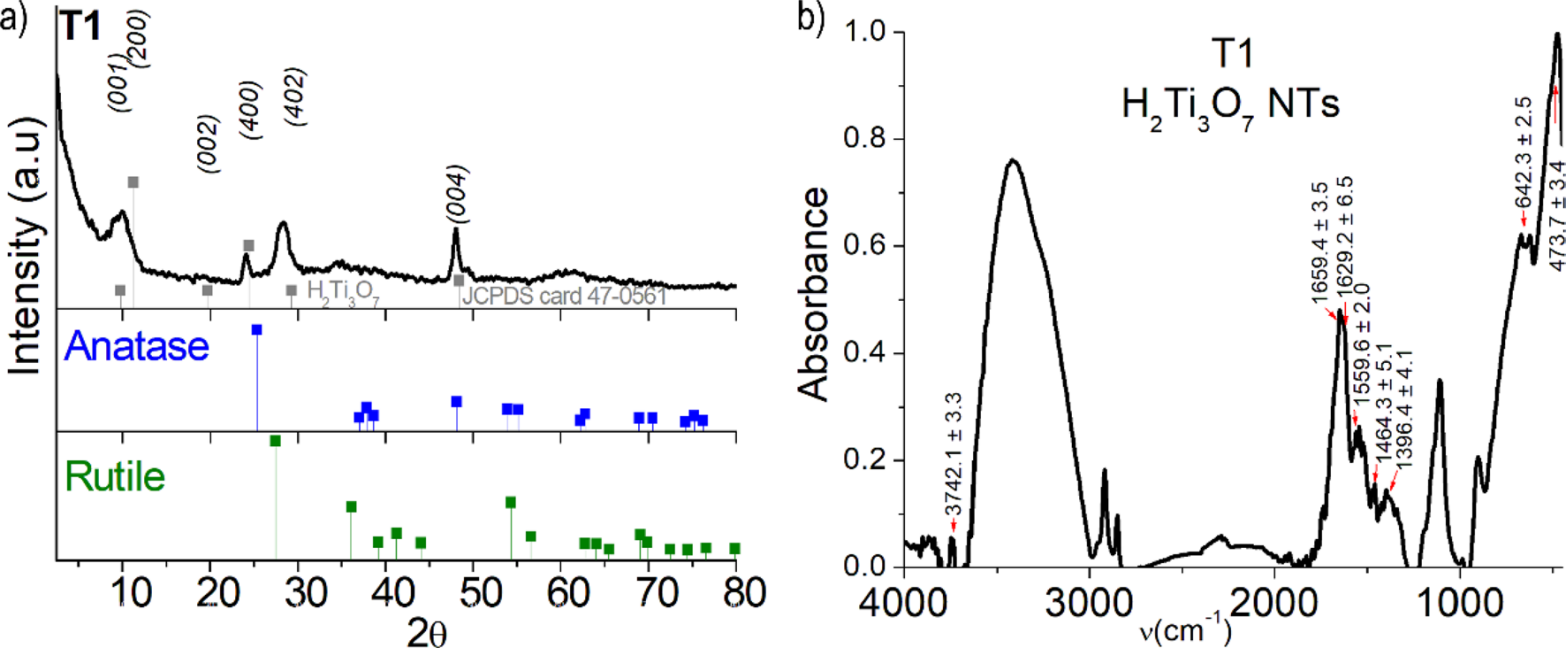
(**a**) X-ray diffraction (XRD) of T1 (black line), H_2_Ti_3_O_7_ patterns (JCPDS card 47-0561) (grey line), TiO_2_ anatase (blue line) (JCPDS card 89-4921), and TiO_2_ rutile (green line) (JCPDS card 89-8304). (**b**) Normalized IR spectrum of T1 in the range of 450–4000 cm^−1^ showing the typical bands of water and Ti/O.

Figure 3a show an X-ray diffraction (XRD) pattern obtained from T1, which agrees with those reported for hydrogen trititanate nanotubes obtained under similar conditions^57,61–63^ as well as to that of titanate bulk reported by Feist et al.^64^ (see Figure S3 and Table S1 in ESI for its corresponding indexation). Slight shifts of the Bragg reflection compared to reference data, in particular that observed at about 2θ = 10° are expected and generally associated with the amount of water intercalated between the tube wall–sheet spaces^65,66^. The nature and content of the water in the sample is in general important for ALD, especially so for the VPM processes where the hydrolysis of the metalorganic precursor is expected to occur with sample–endogenous water; therefore, we have given much attention to the Fourier transform infrared spectroscopy (FTIR) analysis of both pristine nanotubes and their ZnO-functionalized products.

Figure 3b shows the T1 FTIR spectrum in the range 4000–400 cm^−1^. The most important spectral features observed in this spectrum are in line with those previously reported for this compound^64,67^, where absorptions within the lower energy spectral region correspond to the titanate, while absorptions assignable to vibration modes of water or its components dominate the rest of the spectrum. Furthermore, the TiO_2_ NTs spectrum also resemble those reported for films or nanocrystals of anatase exposed to air^68,69^. At low frequencies, we observed an intense, broad, and complex band with a maximum at 473.7 cm^−1^ and a shoulder at 642.3 cm^−1^, which we suggest corresponds to the stretching mode vibrations of terminal Ti–O and bridging Ti–O–Ti bonds, respectively^70,71^. This is in line with the spectra of nanostructured anatase^67^. It is worth noting the relatively large width of this peak, denoting the influence of the medium on the strength of the metal–oxygen bonds in these structures. In general, the hydrated titania surface, as well as the TiO_2_–NTs interfaces, present multiple sites that can interact with the environment by Lewis acid–base interactions, altering the polarity of the Ti–O linkages. The fact that our NTs have both ν(Ti–O) and ν(Ti–O–Ti) near the high-energy limits within these ranges is possibly due to the excess water available in the system. This would enhance both hydrogen bonding with terminal Ti–OH groups and water coordination to cationic sites, diminishing the polarity and increasing the Ti–O bond strength^72^. The simultaneous detection of more than one absorption assignable to each of the internal vibration modes of water –OH-stretching, ν_1_ and ν_3_, and H_2_O-bend, ν_2_– in the T1 FTIR spectrum (see Figures S4–S6, Table S2 in ESI for the deconvolution of different regions of T1 spectrum) indicates the coexistence of several types of water in the sample.

A strong complex absorption feature at 3700–3000 cm^−1^ dominates the OH stretching mode region. The deconvolution of this band (Figures S5 and S6 in ESI) shows that it is formed by at least by four peaks. The positions of the two relatively intense peaks at 3400 and 3230 cm^−1^ point toward the two different forms of water that are like those in bulk water as liquid and ice, respectively^73^. The two minor peaks in this deconvolution would indicate further water types with environments different from those of liquid water or ice. It may be at least partially confirmed by analysing the H–O–H bending absorption bands detected in the 1100–1800 cm^−1^ range (Fig. 3b and Figure S5 in ESI). Water molecule bending vibrations, although less sensitive to coupling with the environment than the ν(OH) ones, are in general relatively narrower single bands^74^. The number of spectral features and relatively large frequency range where they occur clearly reflect the diversity of water types in the sample.

The more prominent feature in this spectral region is the relatively strong and broad band with two maxima centred at about 1659.4 cm^−1^ and 1629.1 cm^−1^, pointing toward to two kinds of water molecules with different H-bonding degrees^68^. The band at 1659.4 cm^−1^, near that of bulk liquid water (1645 cm^−1^)^68^, corresponds to a water type more coupled with H-bonding than that associated with the bending vibration at 1629.1 cm^−1^. This agrees with the IR study of anatase with different degrees of hydration, complemented by ^1^H–NMR–MAS measurements reported by Soria et al.^68^. The authors assigned the band at 1645 cm^−1^ to strong H-bonding coupled to the water bending mode in a multilayer weakly adsorbed to the surface, while they did ascribe the band at 1625 cm^−1^ to a water bending mode in a single layer, fairly strongly adsorbed to the TiO_2_ surface. The shift in water bending IR bands observed for T1 with respect to hydrated anatase^68^ would arise from the different and surely more complex surroundings of water in the H_2_Ti_3_O_7_ lamellar structure. At the red end of the main peak, there is a small broad band where we detected three low intensity peaks centred at about 1559.6, 1464.3, and 1396.4 cm^−1^. Generally, water bending modes near or lower than the frequency of the isolated water monomer (1595 cm^−1^) are indicative of either no H-bonded water molecules or weakly H-bonded water molecules that are coordinated to electrophilic centres^74^. However, this feature has also been reported for hydrated anatase^74^, where the authors assigned an absorption at 1560 cm^−1^ ^75^, appearing as a shoulder at the principal bending band, to strongly adsorbed water interacting with Ti^4+^ sites^75^. The origin of this band was recently corroborated by modelling the interaction of water with TiO_2_ clusters^75^. Calculations showed that the absorption at 1560 cm^−1^ would correspond to the bending mode of a single water molecule coordinated to titanium. Modelling also indicated that such absorption frequency is particularly sensitive to both the water molecule structure and to its interaction with neighbouring oxygen atoms on the TiO_2_ moiety, i.e. on the O(Ti)–H distances and the corresponding dihedral angles^75^. The molecular structure of the HT-TiO_2_ nanotubes is certainly more complex than the TiO_2_ surface; therefore, the multiplicity of the band centred at 1559.6 cm^−1^ can be rationalized as arising from a wide distribution of single water molecules on the surface or inside the nanotube walls.

### Functionalization of H_2_Ti_2_O_7_ by VPM

**Figure 4:**
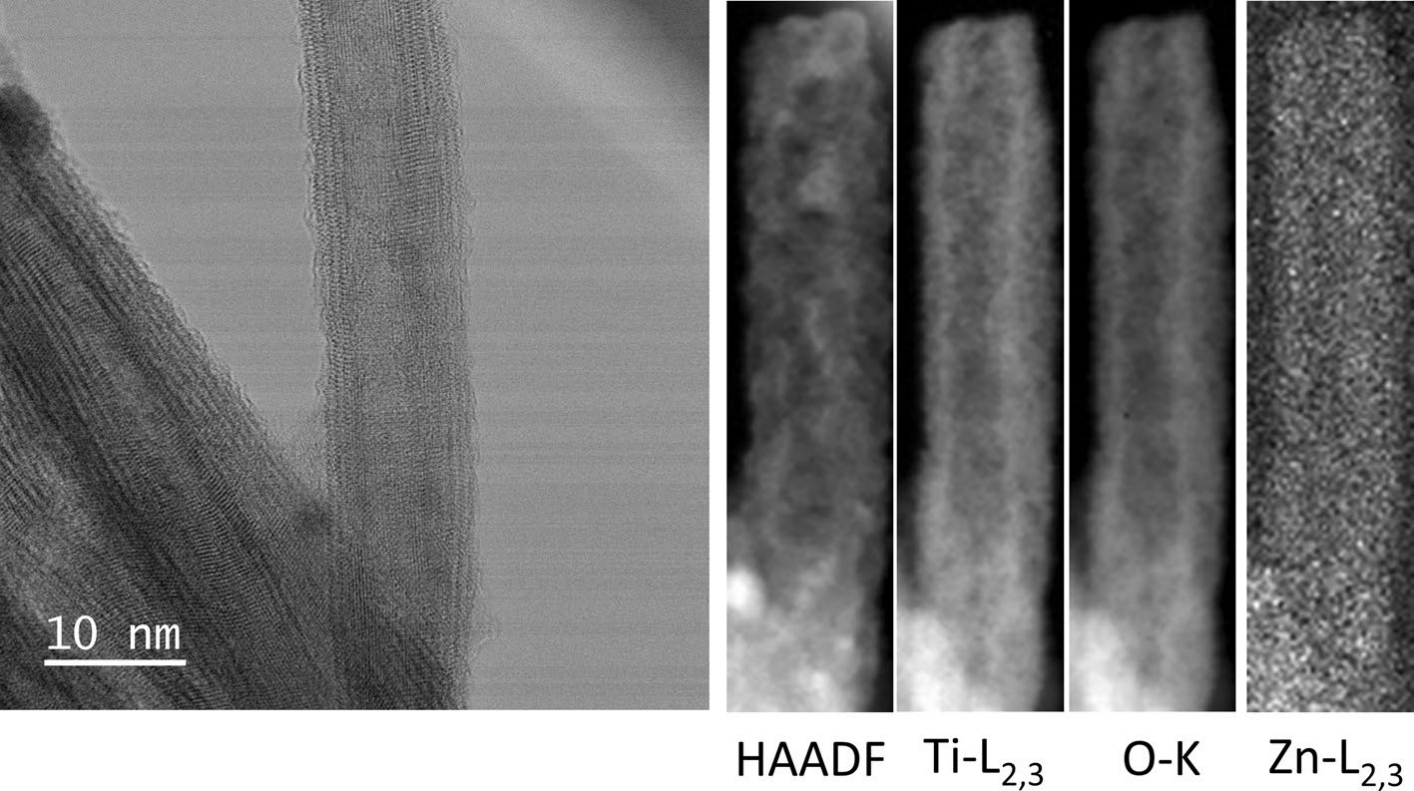
STEM and electron energy loss spectroscopy (EELS) of T2. From left to right, bright field (BF- obtained prior to carrying out the EELS acquisition), high-angle annular dark-field (HAADF); Ti-L_2,3_, O-_K_ and Zn-L_2,3_.

**Figure 5:**
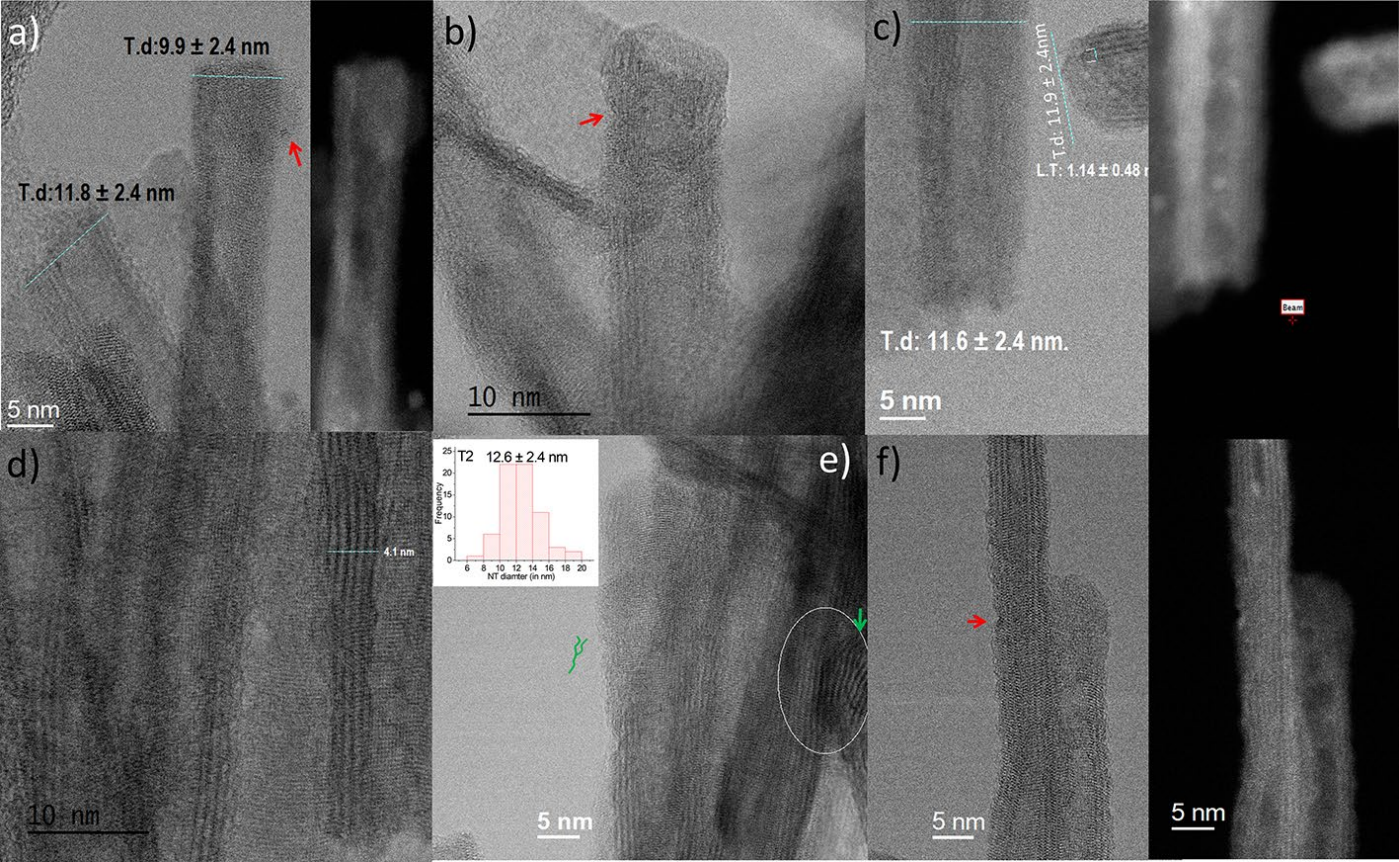
STEM micrographs of T2 showing the widening and waveform of nanotubes (NTs) obtained at 60 kV in Nion Ultra STEM 100MC 'HERMES' (**a**, **c**, **f**) bright field (BF) vs High-angle annular dark-field (HAADF) inset. (**b**, **d**, **e**) BF micrographs. Fracture (red) and dislocation (green).

A scanning transmission electron microscopy (STEM) analysis of the VPM-sample, T2, shows a tubular fibrous product similar to pristine nanotubes indicating that the shape, as well as the laminar nature of the original tubes, is maintained after the metalation: Figs. 4 and 5 show elemental maps produced by electron energy-loss spectroscopy (EELS) spectrum imaging (see “Methods” section). It is observed that the tube wall structure clearly consists of Ti-L_2,3_ and O-_K_, while a very weak Zn-L_2,3_ signal suggestive of the presence of Zn in trace quantities also appears to locate in the walls (the Zn quantity is close to the detection limit approx. 2% in the experimental conditions, making the map extremely noisy, see Figure S7 in ESI for an example of a representative spectrum). This, together with further evidence of the presence of Zn from less localized examination via TEM (see Figures S8–S13 in ESI for representative examples and Figure S14 for energy dispersive X ray (EDX) analysis) would be consistent with a homogeneous, low-concentration ZnO presence throughout the structure. It also reveals that the exposure of H_2_Ti_3_O_7_ to the organometallic reagent produces a noticeable deterioration of the material regularity in terms of tube shapes (see red and green arrows in Fig. 5 and ESI for representative images). The widening of the interlamellar space of some regions of the wall (Figs. 4, 5e,f) leads to a deformation of the external Ti_3_O_7_ layers that impart a wavy appearance to the nanotube surface, and decreases the internal diameter (Figs. 4, 5c–f). These tube distortions make sense considering the complexity of the diffusion mechanism of a reactive gas like DEZ into nanometric spaces, which are chemically, not homogenously active due to different types of confined water within the H_2_Ti_3_O_7_ nanotube walls^68–71,74,75^. However, there are also zones of the sample where it is possible to detect regularity in the distribution of the sheets within the tube walls. As observed in the inset of Fig. 5e, the external diameter of the T2 tubes varied approximately in the range of 12–16 nm, with an average value of ca 12.6 ± 2.4 nm, revealing the T2 NTs external diameter frequency distribution performed by testing 67 NTs. Meanwhile, the average diameter of the internal channels and the tube-wall thickness are 3.0 ± 1.3 and 5.3 ± 2.3 nm, respectively (see Table 1).

Figure 5f shows the HAADF and BF STEM images of a nanotube of greater crystallinity. The structure of the zone between the high contrasted fringes, where many very small particles are apparent, points to the replacement of water molecules in the original nanotubes by intercalated zinc species, Figure S15 in ESI shows abundant nucleated ZnO NPs obtained setting the ALD chamber at RT instead of 120 °C^76^.

**Figure 6:**
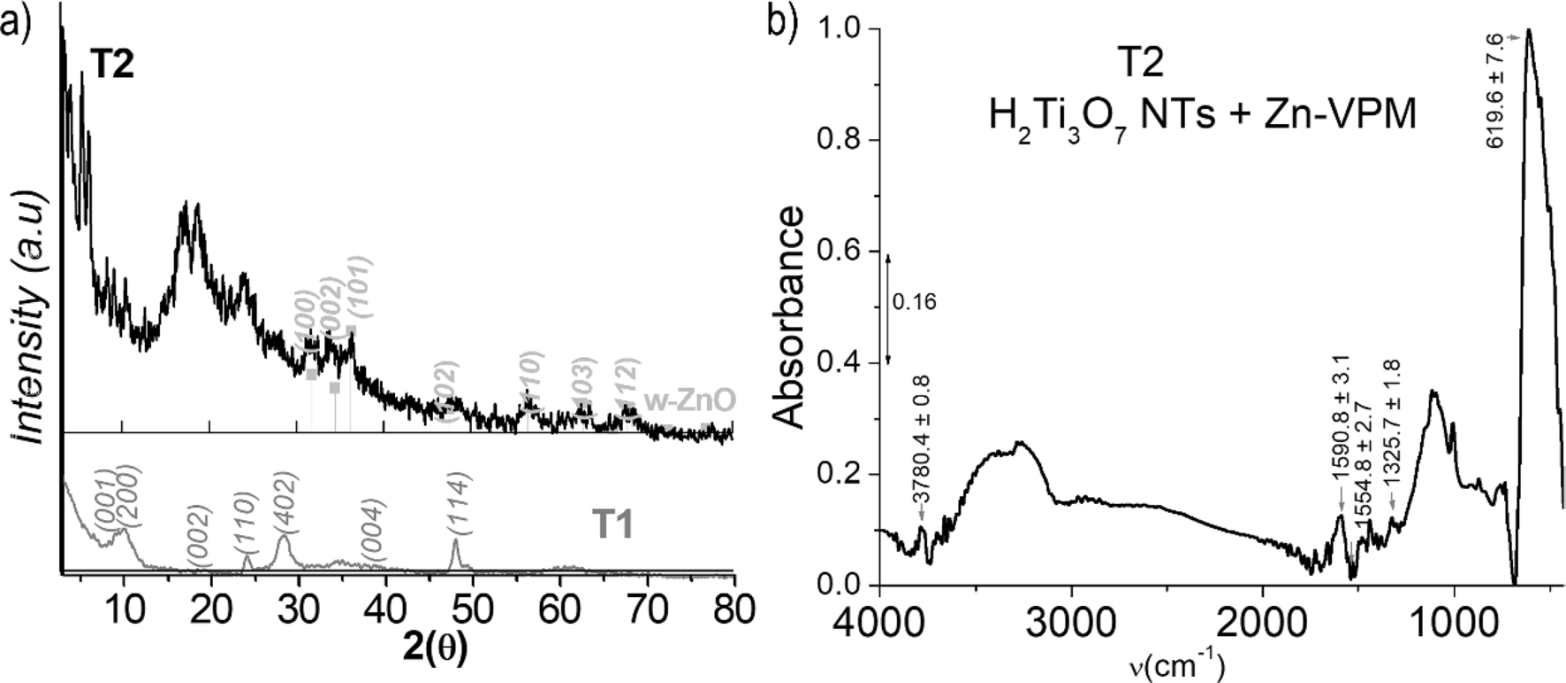
X-ray diffraction of T2 (**a**) contrasted with T1 and ZnO (ICDD card 361451). The XRD analysis of the sample provided knowledge of the chemical nature of the Zn incorporated by VPM and the peculiarities of the laminar wall morphology of the nanotubes. Normalized IR spectrum of T2 in the range of 450–4000 cm^−1^ (**b**).

The XRD patterns of T2 shown in Fig. 6a corroborate the deterioration of crystallinity caused by the Zn-metalation using the VPM process. A significant feature is the clear presence of the three Bragg reflections characteristic of the wurtzite (w) phase of ZnO at 2θ = 31.75° (100), 30.41° (002), and 36.24° (101). The diffraction pattern of the original H_2_Ti_3_O_7_ structure almost disappears with only the reflections at 2θ = 24.19° (400) and 28.4° (402) remaining. The most characteristic XRD peak of H_2_Ti_3_O_7_ normally observed around 2θ = 10° and attributed to diffraction of crystal planes perpendicular to the longitudinal axis of the nanotube, is replaced by a series of reflections characteristic of laminar species, which are observed at smaller angles (Fig. 6a, details in Figure S16 and Table S3 in ESI). In the low 2θ region (2θ < 7°), four sharp peaks assignable to 4 different (001) Bragg reflections corresponding to basal interlaminar distances of 24.45 Å (3.61°), 20.96 Å (4.21°), 16.05 Å (5.50°), and 14.06 Å (6.28°), are found. The corresponding 002 and 003 reflections are detected in the 2θ ranges of 7.5°–13.5° and 15.5°–19°, respectively. This particular peak intensity profile (Fig. 6a) in which the intensity of the even-order reflections is smaller than the odd ones is similar to that previously observed in TiO_2_ or ZnO laminar nanocomposites intercalated with surfactants^77–79^, a phenomenon attributed to double-layer laminar systems^78^. Furthermore, the XRD data suggested that at least one of the causes of the interlaminar distance widening in the nanotube wall is the formation of w-ZnO nanocrystals between the H_2_Ti_3_O_7_ sheets^80^.

The presence of ZnO in the product can be rationalized by the series of reactions described by Eqs. (1)–(3). Most of these processes are triggered by the chemical nature of the organometallic precursor. Given the high polarity of the metal–carbon bond in DEZ, it is expected that it not only adsorbs specifically on, but also reacts with the nucleophilic and electrophilic sites present in the substrate. In the presence of excess organometallic precursor and the absence of water, both types of oxygen atoms present in H_2_Ti_3_O_7_, Ti–O–Ti, and Ti–OH constitute nucleophilic sites potentially capable of interacting with the Zn of DEZ. However, interaction with the acidic groups of Ti–OH is expected to be labile and exergonic, leading to the formation of the Zn^2+^ salt, ZnTi_3_O_7_ (Eq. 1). This reaction is in fact similar to the process of the formation of H_2_Ti_3_O_7_ from alkali tri-titanate Na_2_Ti_3_O_7_, but in the reverse direction. The Zn^2+^ ions in ZnTi_3_O_7_ would be counter ions located in the interlaminar spaces of stacked polyanionic (Ti_3_O_7_^−^)_n_ layers, similar to that reported for many lamellar titanates^61,81^. However, the presence of different groups of water that exist in the nanotubes used in this work (H_2_Ti_3_O_7_·nH_2_O) significantly alters the products of these reactions. Since H_2_Ti_3_O_7_ is a weak Bronsted acid, the ZnTi_3_O_7_ salt (Eq. 1) would rapidly hydrolyse under such conditions by regenerating the acid and producing ZnO (Eq. 2). The presence of water would also produce the interactions of DEZ with the Ti–O–Ti bonds that also underwent hydrolysis, degrading the H_2_Ti_3_O_7_ to ZnTiO_3_ (Eq. 3). The direct hydrolysis of DEZ (Eq. 3) by endogenous excess water cannot be disregarded, particularly by the water fraction located in the central cavity of the nanotubes.

1 $${\text{H}}_{2} {\text{Ti}}_{3} {\text{O}}_{7} + {\text{ZnR}}_{2} \to {\text{ZnTi}}_{3} {\text{O}}_{7} + 2{\text{HR}}$$

2 $${\text{ZnTi}}_{3} {\text{O}}_{7} + 2{\text{H}}_{2} {\text{O}} \to {\text{ZnO}} + {\text{H}}_{2} {\text{Ti}}_{3} {\text{O}}_{7}$$

3 $${\text{H}}_{2} {\text{Ti}}_{3} {\text{O}}_{7} + 3{\text{ZnR}}_{2} + 2{\text{H}}_{2} {\text{O}} \to 3{\text{ZnTiO}}_{3} + 6{\text{HR}}$$

Figure 6b shows the T2 IR spectrum. The complex absorption band observed at lower frequencies, unambiguously attributed in the pristine sample T1 to the stretching modes of Ti–O and Ti–O–Ti bonds, appears in this case as a narrower asymmetric absorption band at 619.6 cm^−1^, blue-shifted with respect to the pristine nanotubes. This band narrowing reveals a series of low intensity and ill-defined features corresponding to water libration modes, which as commented above are expected to appear in the range of 600–900 cm^−1^ ^82^ or the presence of Zn species^83^. Vibrations associated with the Ti–OH bonds in pristine nanotubes are also apparent in the VPM sample. The medium intensity band assigned to the bending mode of Ti–OH centred at 1325.7 cm^−1^ in the nanotubes is observed almost at the same position, while corresponding OH stretching is observed at 3780.4 cm^−1^, slightly blue-shifted with respect to the precursor, possibly due to relative weaker H-bonding or the existence of Zn species^84^.

The most important changes with respect to pristine nanotubes induced by the VPM treatment concern the OH stretching and water bending regions. In the spectrum of the Zn-metalized sample, we also observed a broad complex absorption band with two apparent maxima at about 3280 and 3390 cm^−1^, dominating the OH-stretching spectral region (Fig. 6b). However, the blue shift and relative lower intensity of these bands reveal changes in both the content and the types of water in the sample. In the region of water bending, the spectrum of the VPM sample is quite different from that of the original nanotubes. There is only a well-defined peak of relatively low intensity, centred at 1590.8 cm^−1^ ^71^, red-shifted with respect to that of the starting compound. At higher frequencies, in a region where bending vibrations of water are highly linked to its environment^71^, there is a weak peak at 1633.4 cm^−1^. Interestingly, the band attributed to the bending mode of the water coordinated to Ti^4+^ observed at 1559.6 cm^−1^ disappears completely. Note that the diminution of water (Figures S17–S18 in ESI) qualitatively evidenced in the spectrum agrees with the nature of the VPM process, implying a reaction with DEZ (Eqs. 1–3). A quantitative evaluation of water diminution was unfortunately impossible due to the pre-treatment of the VPM sample.

**Figure 7:**
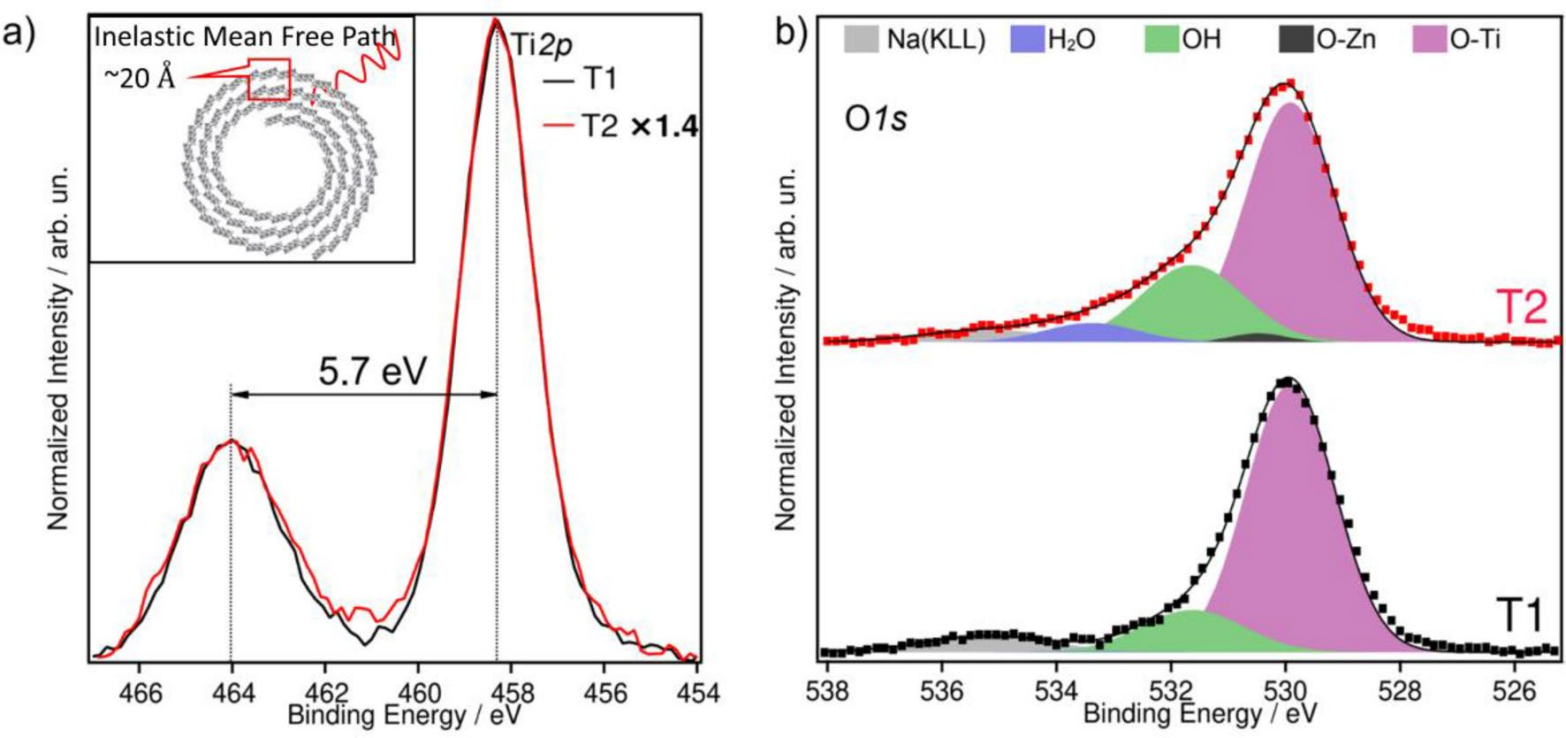
XPS spectra of the Ti2p (**a**) and O1s regions (**b**) for the samples T1 and T2.

Figure 7 shows the X-ray photoelectron spectroscopy (XPS) spectra of the sample T1 compared with sample T2. The composition of sample T1 has been determined as 22% Na, 14% Ti, 50% O and 14% C. The spectrum of the composite sample T2 reveals only a small contribution from Zn, which comes along with a reduction of the Na and Ti concentrations in the near-surface region. For T2 a composition of 2% Zn, 18% Na, 10% Ti, 53% O and 17% C is derived. Figure 7a shows the Ti2p region which depicts a prototypical spectrum for Ti in a 4+ valence state (binding energy (BE) at 458.4 eV and 464.1 eV), identical to what is obtained for TiO_2_^85,86^.

Figure 7a shows that the Ti intensity is decreasing from T1 to T2. This is a consequence of the high surface sensitivity of this method. The inelastic mean free path of the photoelectrons is limited to roughly 20.5 Å (see inset), thus any additional species in the surface near region leads to a damping of the Ti intensity. Taking the pristine nanotubes (sample T1) as a reference, the Ti content at the surface of sample T2 is reduced to 71%. However, no change in the peak shape or its position is observed for the sample T2, indicating that there is no change of the Ti valence state (4+) upon introducing ZnO into the composite. Figure 7b shows the XPS spectra of the O1s region for T1 and T2. For the pristine trititanate nanotubes two contributions to these regions have been identified: O bound to Ti (BE at 529.9 eV) and hydroxide (BE at 531.6 eV). Similarly, as for TiO_2_, H_2_O should dissociatively adsorbs on the NTs in the monolayer, contributing to the OH component in the O 1s spectrum. Multilayers of H_2_O will not be stable on these surfaces under UHV conditions at room temperature^87^.

For sample T2, an additional component arises centred at 530.5 eV binding energy, which agrees with the expected position of O bounded to Zn. Furthermore, another peak at 533.4 eV is observed which has been assigned to molecularly adsorbed water. At the same time, the contribution of the hydroxide component to the spectrum is increased and the intensity of the Ti related O species is damped. In comparison to T1 the O-Ti species is reduced to 88%, which follows the same trend as the damping of the Ti2p core level peak.

The presence of H_2_O in sample T2 is attributed to the adsorption of molecular H_2_O at Zn sites in the ZnO NPs^88,89^. Also, a higher density of surface defects facilitates the H_2_O and OH adsorption in the composite material^87,88^.

### Functionalization of H_2_Ti_2_O_7_ by MPI

**Figure 8:**
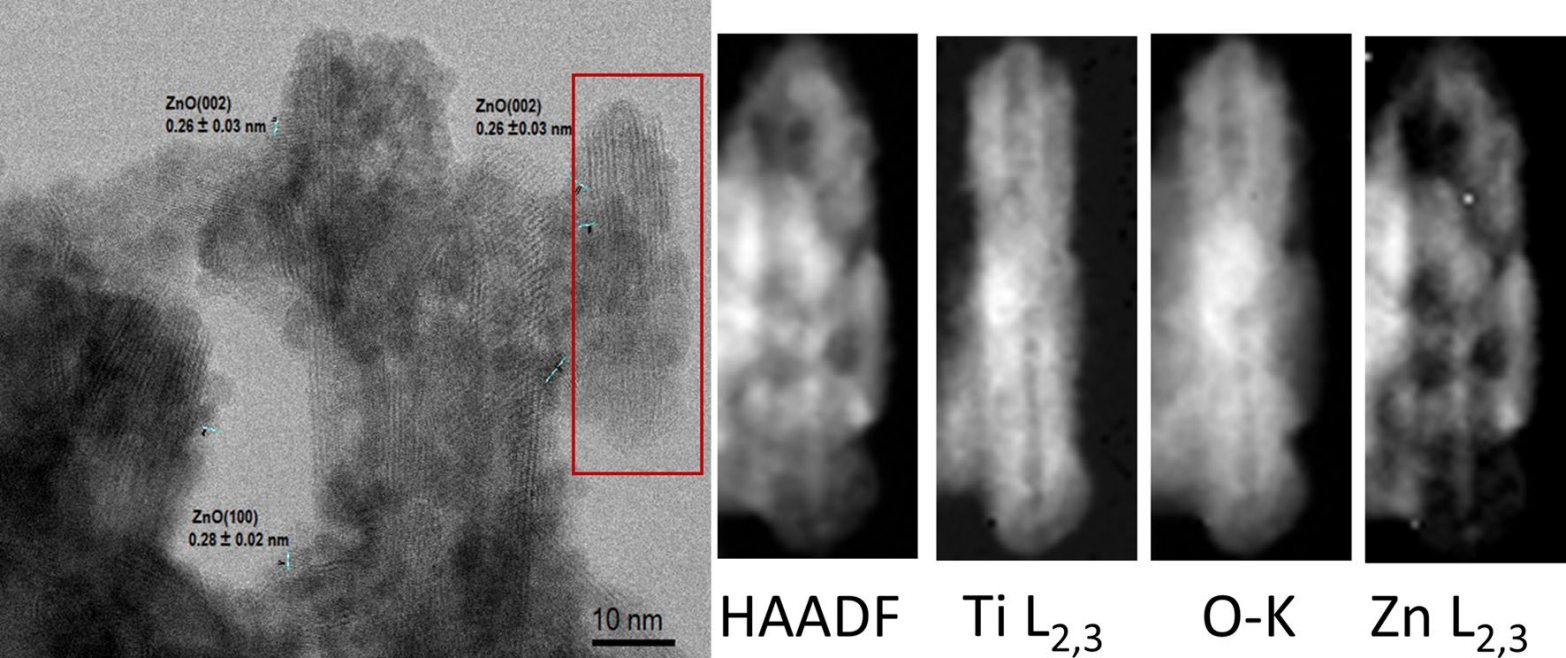
STEM micrography and Electron energy loss spectral (EELS) of T3. From left to right, bright field (BF- obtained prior to carrying out the EELS acquisition), high-angle annular dark-field (HAADF); Ti-L_2,3_, O-K and Zn-L_2,3_ elemental maps.

**Figure 9:**
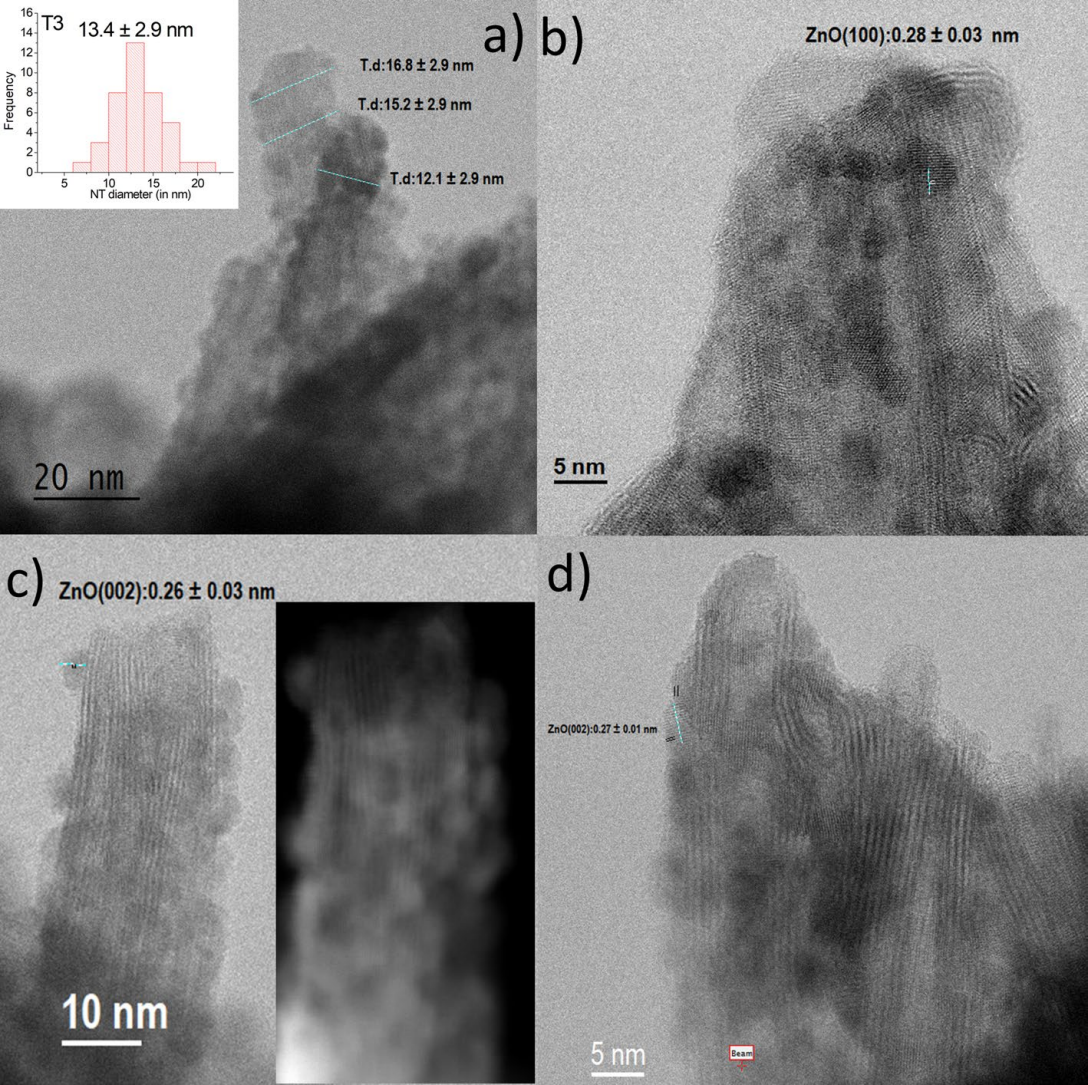
BF and HAADF STEM of T3. T3 STEM micrographs showing widening deterioration, the waveform of NTs, and ZnO crystallographic orientations at 60 kV in Nion Ultra STEM™ 100MC 'HERMES' (a–f). (**a**) BF and histogram of T3 NTs diameter distribution (inset), (**b**) BF (Figure S19 in ESI shows additional STEM/EELS micrograph), (**c**) BF versus HAADF (inset), (**d**) BF.

Figures 8 and 9 show the STEM/EELS analysis of the MPI product, T3, obtained after 10 MPI-cycles, and reveal the formation of a tubular fibrous product (see Figures S7 for EELS spectrum, S19 for additional STEM/EELS micrograph, S20–S23 in ESI for further examples and S24 for energy dispersive X-ray (EDX) analysis). Figure 8 shows a nanotube surrounded by small nanoparticles indicating that the shape and the laminar nature of the original tubes are maintained after the metalation but with a higher effect on the lamellar structuration of Ti_3_O_7_ (see Fig. 9d). Here, despite that Zn quantity increase from T2 to T3 (Zn T2 < T3) and the Ti/O ratio decrease (Ti/O T2 > T3), the Zn is distributed mainly outside of Ti_3_O_7_ NTs, revealing the role of exogenous and endogenous waters during the MPI and VPM processes and, highlighting the difference between both mechanism of metalation.

Figure 9a–d shows that both the crystallinity and the morphological regularity of the observed structures are vastly inferior to those of T1. That notwithstanding, it can be clearly seen that the sample is mainly composed of tubular species partially decorated with relatively large ~ 1.5 nm, globular particles distributed randomly upon their surface. A qualitative analysis of the lattice fringe spacings observed in Fig. 9a,b (0.28 ± 0.02 nm and 0.26 ± 0.03 nm) agree well with expected the (100) and (002) crystallographic planes spacing of ZnO, respectively, being the nearest H_2_Ti_3_O_7_ crystallographic plane (300) at 0.318 nm (see XRD Table S3 in ESI). The inset of Fig. 9a shows the frequency distribution of the outer diameters of T3 NTs performed by testing 43 NTs (Table 1). The result exhibits a major frequency of tubes with outer diameters in the range of 11–14 nm, with an average of about 13.42 ± 2.89 nm.

Although the further deterioration of the nanotubes using the MPI process (DEZ and exogenous H_2_O) is not expected, Fig. 9c,d reveals the extreme impact of this process in the Ti_3_O_7_ lamellar structuration.

**Figure 10:**
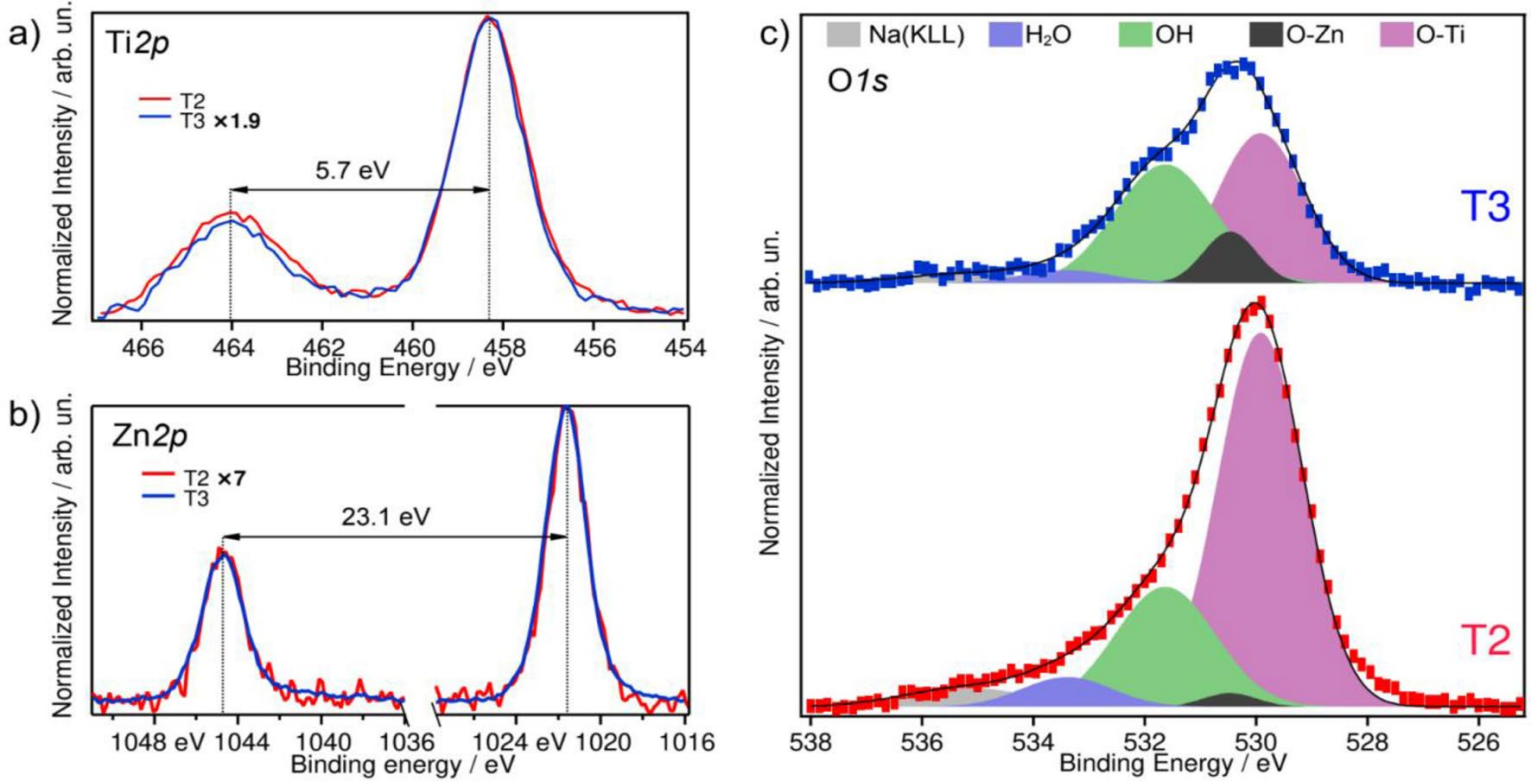
XPS spectra of the Ti2p (**a**) and O1s regions (**b**) for the samples T2 and T3.

Figure 10 shows the comparison of the XPS spectra of T3 and T2. The spectrum of the composite material prepared by the MPI process exhibits a strong increase in Zn content at the surface resulting in an overall composition of 15% Zn, 10% Na, 7% Ti, 50% O and 17% C. Figure 10a depicts the Ti2p core level region. Despite the higher quantity of Zn introduced in T3, the Ti remains in the 4+ valence state but decreases to 37% as compared to the pristine NTs. Figure 10b shows Zn2p regions for both samples (BE at 1044.39 eV and 1021.8 eV), it indicates that the increase in the Zn concentration is homogeneous throughout the surface near region for both samples. The valence state of Zn can be derived from an Auger parameter analysis in which the Zn 2p core level and the Zn LMM Auger line enter. The sum of the photon energy and the Auger parameter is 2009.9, which is in perfect agreement with the tabulated values for ZnO indicating a 2+ valence state of Zn^85^. For metallic Zn a value of 2014 would be expected.

Figure 10c shows the XPS spectra of the O1s region for the two samples. In the spectrum from T3 the component related to Ti-bonded O species further decreases in intensity. In comparison to the pristine NTs, the damping amounts to 37%, similar as reported for the Ti2p core level above. The intensity of the Zn related O peak increased by a factor of 3.7 as compared to T2. This enhancement is smaller as expected from the Zn2p and Zn(LMM) intensities. The H_2_O peak is reduced by a factor of two in comparison to T2, while the hydroxide related peak remains unchanged in intensity, reinforcing the idea of the existence of water clusters in the samples. On the other hand, the absence of metallic Zn concluded from the Auger parameter and a constant C1s contribution from adventitious carbon throughout all samples (see Figure S25 in ESI) allow to discard the presence of unexpected bonds like Zn–Zn and Zn–C, respectively.

**Figure 11:**
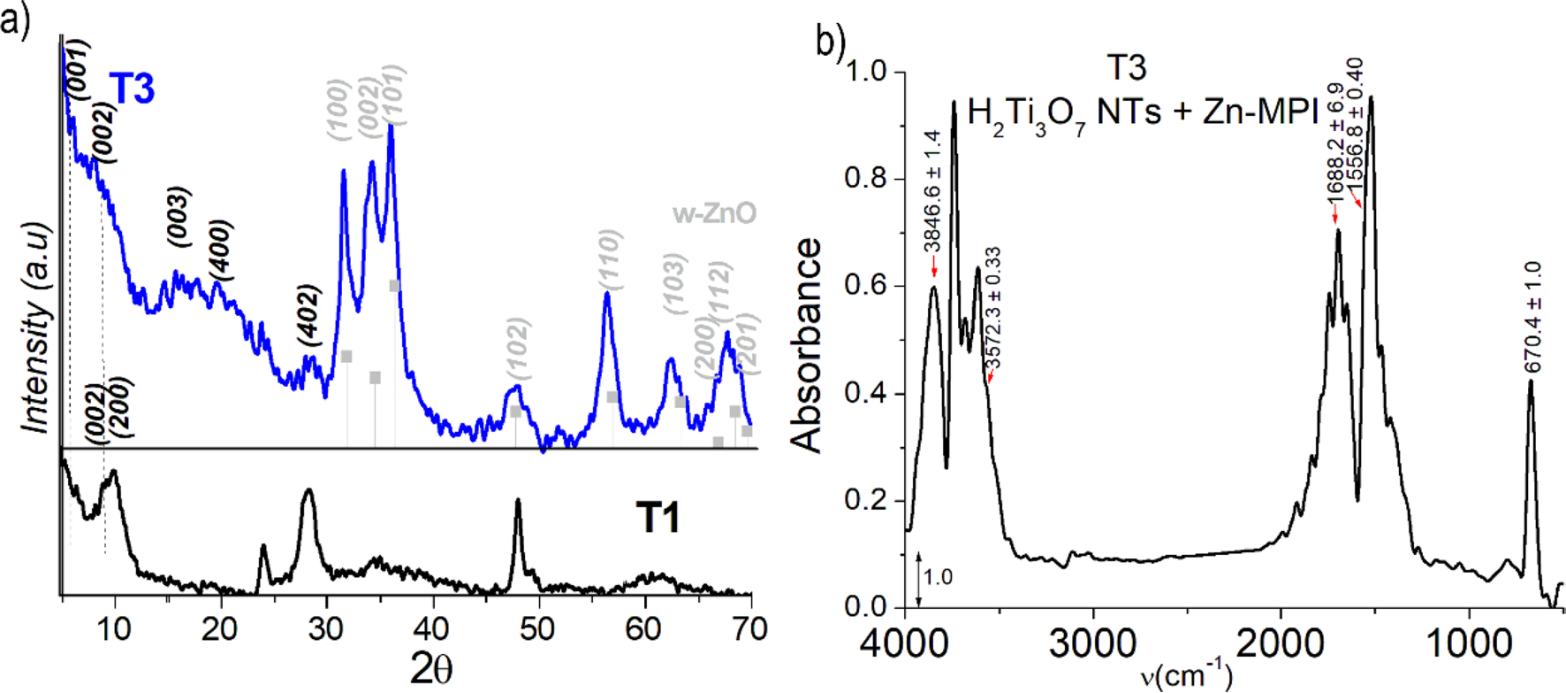
X-ray diffraction of T3 (**a**) contrasted with T1 and ZnO (ICDD card 361451). And Normalized IR spectrum of T3 in the range of 500–4000 cm^−1^ (**b**).

Figure 11a show an XRD pattern obtained from T3. The crystallinity of the T3 product is relatively poor; however, at first glance its XRD pattern seems to correspond mainly to the overlapping of Bragg reflections of both w-ZnO and original nanotubes, including three of the five characteristic diffraction peaks of pristine nanotubes. Namely, peaks near the nanotubes; peculiar reflections at 2θ = 10.36° (200), 20.72 (400), and 28.3° (402). Moreover, there is a cluster of reflections of relatively low intensity in the low-angle region of the diffractogram, which is similar to that discussed for sample T2 and that could be attributed to a set of structures with different interlaminar separations (Figure S26 and Table S3 in ESI). The probable presence of by-products from the reaction of the nanotubes with DEZ with excess water, such as zinc titanates^12,90^ and small zinc hydroxides crystallites^80^, leading to multiple low intensity reflections in areas not obscured by diffraction signals of the more crystalline w-ZnO, could be also contributing to the complexity of this diffractogram.

Figure 11b shows the FTIR spectrum of T3 Well-defined sharp spectral features significantly differ from the relatively broad spectral bands in samples T1 (Fig. 3b) and T2 (Fig. 6b) analysed previously. In the region of the Ti–O and Ti–O–Ti stretching modes we observed for T1, a unique sharp middle-intensity peak at 642.3 cm^−1^ occurred. Similar to the VPM sample, we suggest that the narrowing and red-shift of the v(Ti–O) peak is due to the weakening of the Ti–O–Ti vibrations modes, which totally disappear in this case. This effect seems associated with the loss of structural integrity of the tube walls, which reaches a maximum in the MPI metalation. From such a correlation, we could further infer that the terminal Ti–O oscillators are at both the nanotube surface and the intra wall layers, while the Ti–O–Ti absorptions observed in this frequency range would result from any cooperative effect induced by the multiwall nanotube structural nature. Thereafter we can explain the strength in intensity of the water absorptions peaks observed in the MPI sample spectrum (Fig. 11b). In the case of highly degraded nanotube walls, the availability of terminal Ti–O oscillators is in practice limited to a monolayer of space separating Ti–OH groups distributed upon the tube surface, thus with much more room remaining for the adsorption of one or possibly more water layers.

The shape of the T3 spectrum in the bending mode region has characteristics that point to the presence of surface water. The wide band centred at 1688.2 cm^−1^ in T3 seems to correspond to the superposition of absorptions from several slightly different types of water. In general, relatively high-water bending frequency values arise from surface states of water hydrogen bonded to a substrate, which occurs, for example, on the surface of ice. The study of “free” water molecules isolated in a D_2_O matrix^72^ using different experimental techniques, such as difference spectra^91^ or more recently sum-frequency generation (SFG)^75,82^, or through theoretical simulations^75,82^, showed that there exist three types of vibrational modes for water located on the surface, differing in the saturation degree of the four labile coordination centres or dangling bonds of the molecule. Namely, tri-coordinated water with either a dangling H (d–H) or a dangling O (d–O), both on the surface layer, and tetra-coordinated water (s–4) on second and following sublayers. The bending mode frequency of water on the surface of ice nanoclusters at 10 K follows the order d–H (1650 cm^−1^) < s–4 (1675 cm^−1^) < d–O (1690 cm^−1^)^73^. Assuming the same tendency, the absorption band centred at 1688.19 cm^−1^ would indicate a surface dominated by water molecules with their oxygen atoms exposed outwards. On the other hand, the band at 1556.8 cm^−1^ is near that at 1559.6 cm^−1^ for sample T1 or at ca. 1560 cm^−1^ in hydrated anatase^75^, which corresponds to water coordinated to centres strongly electrophilic for Ti^4+^.

The series of relatively sharp bands in the OH stretch spectral region observed for sample T3 (Fig. 11b), where the characteristic signal broadening for hydrogen bonding coupling is virtually non-existent, are rather atypical. Beside the ν(OH) bands from the different types of water in sample T3, in this region we expect to detect the absorptions from the hydroxides of Ti and Zn produced by the MPI process (DEZ/H_2_O). In line with FTIR studies of the surface of hydrated titania^68^, we suggest that the band at 3743.5 cm^−1^ in the spectrum of sample T3 corresponds to the stretching mode of the terminal Ti–OH group. Furthermore, the OH stretching band in the Zn–OH group reported for the hydroxylated ZnO crystal at 3572.3 cm^−1^ ^92^ appears as a shoulder at about the same frequency in the T3 sample spectrum.

This range does not show any assignable absorption to the 2 × ν2 bending overtone. The ice surface modes in the spectra mentioned above correlate inversely with their corresponding bending modes according to the empirical bending/stretching Falk´s relationship^91^ (Figures S27 and S28 in ESI). Considering this, we suggest the band observed at 3679.4 cm^−1^ corresponds to the O–H stretch-mode vibration of surface d–O water molecules (ν_2_ = 1695 cm^−1^) discussed above. Analogously, we assign the vibration ν(OH) at 3846.6 cm^−1^ to the bending mode of water molecules coordinated to the nucleophilic centre Ti^4+^ at 1556.8 cm^−1^.

The water spectrum of the T3 sample discussed above is quite peculiar. The positions of the predominant peaks are similar to those found for water in the condensed state, while their relatively narrow band shapes point to a reduced hydrogen bonding network. We suggest that the origin of this apparent anomaly arises from a discrete distribution of the water adsorption centres upon the substrate, probably M–OH groups, far enough apart to avoid an extensive water aggregation which would lead to isolated molecules and small water clusters. Finally, it is noteworthy that the MPI treated TiO_2_ NT sample is, to the best of our knowledge, the first example of a material where it is possible to detect surface water vibration modes by conventional FTIR spectroscopy under normal conditions.

The comparative FTIR analysis of the H_2_TiO_7_ nanotubes and their products functionalized with ZnO through VPM and MPI effectively complements the results of this report. On the one hand, it allowed us to corroborate the structural and morphological observations of the products and, on the other, to visualize the importance of the endogenous and exogenous water involved in the ALD-type processes used. In both processes, a dehydration of the sample occurs. Among the different types of water available in the pristine nanotubes, the VPM mainly affects the water coordinated to the Ti^4+^ sites, leading to the formation of ZnO in the tube–walls interlaminar spaces, whilst superficial and interstitial water induces a slight degradation of the multi-wall structure of the tubes. However, the organometallic Zn precursor under water excess (MPI) seems to act mainly on the tubular walls, leading almost exclusively to coated nanotubes on whose surfaces water reappear coordinated to metallic centres, such as those observed in hydrated anatase surfaces. These results open the way to designing strategies to obtain new layered metal oxide nanocomposites by managing variables, such as the degree of substrate hydration, work temperature, or water pulse-time in the ALD-type treatments, which were not investigated in this work.

**Figure 12:**
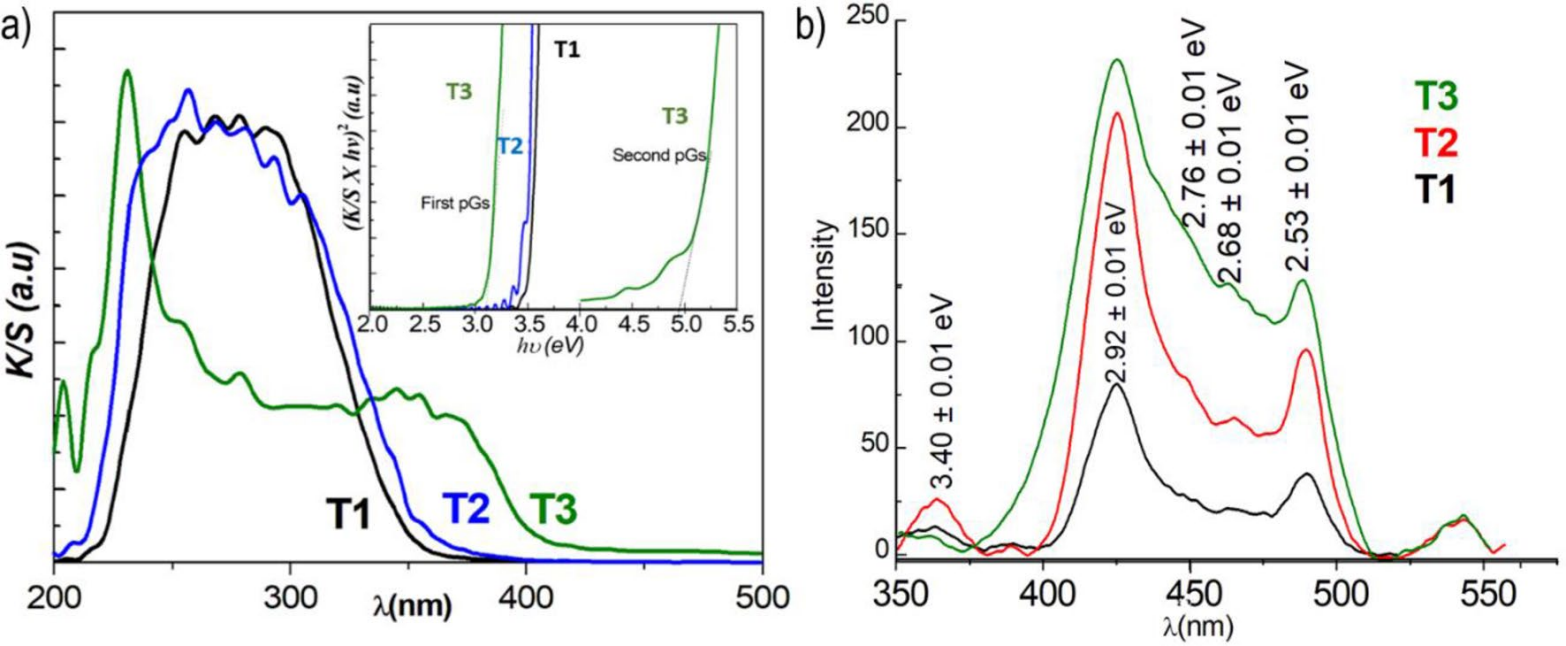
Reflectance diffuse spectra (after Kubelka–Mulk transformation)^93^ of T1, T2, and T3 lamellar H_2_Ti_3_O_7_ NTs, and (inset) plot (K/S × hν)^2^ against hν showing the linear fitting to the main linear segment of the curve considering a direct band gap transition., according to the Tauc equation (**a**). Luminescence emission spectra of T1, T2, and T3 (**b**).

Figure 12a shows the absorption spectra obtained from the corresponding diffuse reflectance spectra using the Kubelka–Mulk transforms^93^. Interestingly, the spectra of the pristine semiconductor (anatase) and those of the used H_2_Ti_3_O_7_ nanotubes, as well as those subjected to the VPM treatments, are qualitatively similar. That notwithstanding, we also clearly observe that the absorption band edge in the product blueshifts compared to bulk TiO_2_. This indicates that the electronic band structure of primitive TiO_2_ remains mostly unaltered, besides the band gap increase expected for a layered semiconductor with two-dimensional confinement^93^. Contrasting with sample T2, the absorption spectrum of sample T3 appears dominated by the presence of ZnO. It is known that the ZnO absorption band edge is strongly affected by particle size^94^. The latter is reflected in the spectrum of the T3 sample where we mostly observe two prominent features, a wide band with a maximum at ca. 368.50 nm and a sharp one at 230.80 nm. The absorption band edge corresponds to a bandgap of 3.08 eV (Fig. 12a, Table 2) that is within the range of that reported for bulk ZnO^78^, whilst the band at high energies would correspond to the absorption of particles with very small sizes (< 1.5 nm)^94^.

**Table 2:** Optical bandgap energy (Eg) of samples T1, T2 and T3 and related precursors.

Sample	Eg (eV)
TiO_2_ anatase^93^	3.25 ± 0.04
H_2_Ti_3_O_7_ (bulk)^95^	3.36
T1	3.55 ± 0.10
T2	3.40 ± 0.01
T3	3.05 ± 0.14
ZnO (commercial)^78^	3.21 ± 0.03

Figure 12b shows the luminescence spectra of pristine nanotubes and VPM and MPI products. The most prominent spectral features are emissions centred at approximately 425, 463, and 488 nm. All of these emission peaks are near those reported for anatase TiO_2_^93^, that is known for having a low dependence on the size of the nanostructures^93^. Contrasting with its absorption spectrum, the emission spectrum of the T3 sample appears to be dominated by the TiO_2_ nanotubes luminescence emissions. In this spectrum, the characteristic bands of ZnO are not detected, such as the green emission at ca 550 nm^78^. Only some shoulders are observed in the region 510–540 nm, which could be attributed to this oxide. Interestingly, the PL spectra (Fig. 12b and Table S5 in ESI) indicates that the small band around 449 nm in the T1 and T2 spectra are attributed to the exciton emission of TiO_2_, which disappears in the T3 spectrum. The intensity differences of the PL spectra of the three samples, T3 > T2 > T1, correlated inversely with the degree of crystallinity and/or the integrity of the nanotubes discussed above.

The behavior of samples T2 and T3 in the photodegradation of methylene blue (MB) (Scheme S1 and Text on page 1 in ESI) corroborates that the semiconductor properties of both oxides are preserved after ALD-type treatments. However, the photocatalytic activity caused by the synergy between the components of the sample depends on the nature of the ALD treatment, which is practically null in our case for VPM-T2 but clearly improved for the MPI-T3 sample (Figure S29 in ESI). A feature that is in line with previous reports on the effect of semiconductor disposition on photoactivity in TiO_2_/ZnO systems^29^.

## Conclusion

The suitability of ALD techniques, VPM and MPI, for developing titanium dioxide-based semiconductor heterojunctions was explored using layered hydrothermally prepared H_2_Ti_3_O_7_ nanotubes as the model substrate and DEZ as the metal precursor. The VPM process leads to w-ZnO by hydrolysis of the organometallic DEZ with endogenous water available in the substrate, H_2_Ti_3_O_7_·nH_2_O, where there are different types of water—superficial, structural, confined in hydrates, and coordinated to Ti^4+^ centres. ZnO formation mainly occurs inside the tube walls' interlaminar spaces, preferentially consuming the water coordinated to the titanium cations, with the oxide intercalated between the tube walls layers remaining. The VPM organometallic precursor, besides saturating the layer Ti–OH groups, also partially reacts with the tube Ti–O–Ti network, leading to a certain degradation of the nanotube walls. The functionalization of the nanotubes through the MPI process, characterized by great water availability, generates a drastic tube wall demolition, leading to single-wall tubular tritanate species. The ZnO in these nanostructures appears in a bi-modal crystallization as sub-nanometric particles coating the tube surface and as larger particles that are ca. 2 nm in diameter, decorating the tube externally. In both VPM and MPI processes, metalation induces a notorious substrate dehydration that is even greater when using exogenous water (MPI). The optical and photocatalytic (Figure S29 in ESI) properties of the products agree with their expected semiconductor behaviour. The results communicated here constitute a proof-of-concept of the potential of ALD techniques, such as VPM and MPI, for easily tuning product properties by regulating substrate water content, thus appearing as a promising tool for developing tailor-made layered semiconductor materials.

## Methods

### Synthesis of hydrothermal H_2_Ti_3_O_7_ nanotubes (T1)

**Figure 13:**
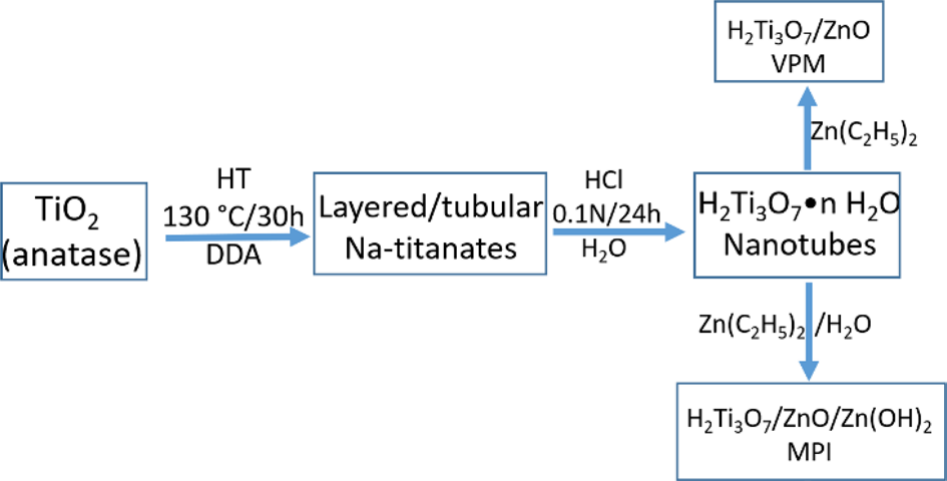
Scheme for the hydrothermal synthesis of titanium oxide nanotubes and their functionalization using VPM and MPI techniques.

Figure 13 shows a scheme for the hydrothermal synthesis of titanium oxide nanotubes and their functionalization using VPM and MPI techniques. About 0.4 g of anatase and 0.4 g of dodecylamine (DDA) were dissolved into 5 mL of 10 N NaOH. The obtained mixture was then moved to a Teflon lined stainless steel autoclave. The reactor was placed in an oven at 130 °C for 30 h and then naturally cooled to room temperature. The obtained solid was treated with a solution of 0.1 N HCl for 24 h, separated, washed several times with deionized water, and dried at 130 °C for 24 h. Finally, the product was pulverized in an agate mortar and preserved in sealed glass jars^96,93^. All the chemical reagents used here were purchased in Sigma-Aldrich; Titanium (IV) oxide, anatase powder, 99.8% trace metals basis, CAS Number 1317-70-0, dodecylamine (DDA) 98%. CAS Number 124-22-1, sodium hydroxide reagent grade, ≥ 98%, pellets (anhydrous), CAS Number 1310-73-2. It is noteworthy to mention that anatase disaggregation in a strong alkaline aqueous solution under relatively mild hydrothermal conditions (110–190 °C, 0.5–3 days) followed by an acidification, leads to nanocrystalline, stoichiometric, tubular products with a global formula of H_2_TiO_3_·nH_2_O. In general, both the almost quantitative reaction yield and the morphology characteristic of the tubes are not very sensitive to changes in the reaction conditions. The products are almost the same, even when using different titanium sources, such as anatase, rutile, or metallic titanium^96^; diverse temperatures (110–190 °C)^64^ and reaction times (0.5–3 days)^63,97^; different acid concentrations^62^; or even when the reaction is performed in the presence of a surfactant^93^.

### Vapor phase metalation (VPM) of H_2_Ti_3_O_7_ NTs (T2)

About 15 mg of H_2_Ti_3_O_7_ NTs suspended in 500 µL of ethanol were homogeneously dispersed on a four-glass slide, which was then dried in air and placed in the ALD chamber (Savanah-100, Cambridge Nanotech Inc and GemStar-6™ by ARRIDIANCE). The metalation was carried out at 120 °C, performing a sequence of 100 VPM cycles (half cycle of ALD and longer purging time) using DEZ as the metal source and argon as the carrier gas. Diethylzinc (DEZ) CAS Number: 557-20-0 (Sigma-Aldrich, product-number: 256781, ≥ 52 wt% Zn basis, LOT number: SHBG8866V), H_2_O (Milli-Q water), Argon (Air Liquid, 99.99% at 40 sccm) and purging gas (100 sccm). Each cycle of VPM typically consisted of the following 3 steps: (i) DEZ pulses of 0.3 s, (ii) exposure times of 40 s, and (iii) purging times of 80 s were used. Samples obtained by this method are hereafter mentioned as T2.

### Multiple pulsed vapor phase infiltration (MPI) of H_2_Ti_3_O_7_ NTs (T3)

H_2_Ti_3_O_7_ substrate samples identical to those described above and using the same ALD equipment were sequentially treated with two ALD precursors, DEZ and water, but with longer purging times (MPI). Ten MPI cycles were performed, where each cycle typically involved the following 6 steps: (i) a DEZ pulse of 0.3 s, (ii) an exposure time of 40 s, (iii) purging for 80 s, (iv) a water pulse of 0.1 s, (v) an exposure time of 40 s, and (vi) purging for 120 s. Samples obtained by this method are hereafter mentioned as T3.

### Characterization

The products were characterized by XRD analysis (SIEMENS D-5000, Cu-Kα radiation), Fourier transform infrared spectrometry (FT-IR, Bruker Vector 22) at a spectral resolution of 4 cm^−1^ (KBr pellet), and transmission electron microscopy (TEM) (JEOL JEM-2011 at 200 kV, JEM 1010 at 100 kV, FEI Tecnai F20 at 200 kV, TALOS G-2 TEM at 200 kV and FEI QUANTA 200 FEG at 30 kV).

Additional scanning transmission electron microscopy was carried out on a Nion UltraSTEM100MC, operated at 60 kV. The optics were configured for a 0.1 nm probe with 31 mrad convergence semi-angle and 20 pA of current. Bright field (BF) and high-angle annular dark field (HAADF) detector angular ranges were calibrated as 0–9 mrad and 92–190 mrad, respectively. Electron energy loss spectra (EELS) were recorded using a Gatan Enfinium ERS spectrometer, with a 44 mrad acceptance half-angle. Dispersions of 0.5 eV/channel or 0.6 eV/channel were used to capture an energy range spanning the Ti L_2,3_ (onset at 456 eV), O K (532 eV), and Zn L_2,3_ (1020 eV) edges simultaneously. EELS maps were generated by integrating each edge over a suitable energy window after removal of the continuous decaying background using a conventional power law function. The data was processed using Principal Component Analysis to remove noise prior to generating the EELS maps, as implemented in the MSA plugin for Digital Micrograph (available commercially from HREM Research https://www.hremresearch.com/index.html). The study of the thermal behavior of T1 was carried out by thermogravimetric analysis (TG) in a TA instrument model Q500. The analysis of 1 mg of T1 was realized at 5 °C/min heating rate under a constant stream of nitrogen at a flow rate of 40 mL/min.

The diffuse reflectance UV–Vis spectra (Shimadzu UV–Vis model 2450 PC spectrophotometer with an integrating sphere) were recorded at room temperature in the range of 200–800 nm at medium scan rates and with a 0.1 nm slit using barium sulphate as the reference. The reflectance measurements were converted to absorption spectra using the Kubelka–Munk function. Luminescence analysis was performed using a Perkin-Elmer spectrofluorometer model L55 with a 150 W xenon lamp.

X-ray photoelectron spectroscopy (XPS) using an AlKα source at a photon energy of 1486.6 eV was employed at a pressure below 2 × 10^−7^ Pa to investigate the surface chemical composition of the samples. The nanotube samples T1–T3 have been dispersed onto a Ta plate. To compensate for charging effects, all binding energy values were calibrated by fixing the C1s core level binding energy at the 284.8 eV. All spectra have been normalized to the intensity average between 810 and 790 eV of the survey scan, respectively. The detail scans presented here have been derived from the normalized spectra by Shirley background subtraction^98^.

## Supplementary Information

Supplementary Informations.
