# Publisher Correction: ZnO nucleation into trititanate nanotubes by ALD equipment techniques, a new way to functionalize layered metal oxides

**DOI:** 10.1038/s41598-021-92227-7

**Published:** 2021-06-14

**Authors:** Mabel Moreno, Miryam Arredondo, Quentin M. Ramasse, Matthew McLaren, Philine Stötzner, Stefan Förster, Eglantina Benavente, Caterina Salgado, Sindy Devis, Paula Solar, Luis Velasquez, Guillermo González

**Affiliations:** 1 Universidad SEK, Instituto de investigación Interdisciplinar en Ciencias Biomédicas SEK (I3CBSEK), Facultad Ciencias de la Salud, Fernando Manterola 0789, Providencia, Santiago Chile; 2 Max Planck Institute of Microstructure Physics, Weinberg 2, 06120 Halle, Germany; 3 Facultad de Ciencias, Universidad de Chile, Las Palmeras, 3425 Nuñoa, Santiago Chile; 4 Queen’s University Belfast, University Rd, Belfast, BT7 1NN UK; 5 SuperSTEM Laboratory, STFC Daresbury Campus, Daresbury, WA4 4AD UK; 6 School of Chemical and Process Engineering, University of Leeds, Leeds, LS2 9JT UK; 7 Living Systems Institute, University of Exeter, Exeter, EX4 4QD UK; 8 Martin-Luther-Universität Halle-Wittenberg, Halle, Germany; 9 Departamento de Química, Facultad de Ciencias Naturales, Matemática y Medio Ambiente, Universidad Tecnológica Metropolitana, Santiago, Chile; 10 Programa Institucional de Fomento a la Investigación, Desarrollo E Innovación (PIDi), Universidad Tecnológica Metropolitana, Santiago, Chile

Correction to: *Scientific Reports* 10.1038/s41598-021-86722-0, published online 08 April 2021

The original version of this Article contained an error in Affiliation 1, which was incorrectly given as ‘Instituto de investigación Interdisciplinar en Ciencias Biomédicas SEK (I3CBSEK), Facultad Ciencias de la Salud, Universidad SEK, Fernando Manterola 0789, Providencia, Santiago, Chile.’ The correct affiliation is listed below:

Universidad SEK, Instituto de investigación Interdisciplinar en Ciencias Biomédicas SEK (I3CBSEK), Facultad Ciencias de la Salud, Fernando Manterola 0789, Providencia, Santiago, Chile.

Furthermore, the authors Sindy Devis, Paula Solar and Luis Velasquez were incorrectly affiliated with ‘Max Planck Institute of Microstructure Physics, Weinberg 2, 06120 Halle, Germany’. The correct affiliation is listed below.

Universidad SEK, Instituto de investigación Interdisciplinar en Ciencias Biomédicas SEK (I3CBSEK), Facultad Ciencias de la Salud, Fernando Manterola 0789, Providencia, Santiago, Chile.

The original Article and accompanying Supplementary Information file have been corrected.

