# Supplementary material for: ZnO nucleation into trititanate nanotubes by ALD equipment techniques, a new way to functionalize layered metal oxides
Source: Sci Rep. 2021 Apr 8;11:7698. doi: 10.1038/s41598-021-86722-0 (PMC8032785; doi:10.1038/s41598-021-86722-0)
Supplement: Supplementary file 1 — Supplementary Informations. [file 41598_2021_86722_MOESM1_ESM.docx]

SREP-19-27008B

**ZnO nucleation into trititanate nanotubes by ALD equipment techniques, a new way to functionalize layered metal oxides**.

Mabel Moreno^1,2,3*,^ Miryam Arredondo^4^, Quentin M. Ramasse^5,6^, [Matthew McLaren](http://pure.qub.ac.uk/portal/en/persons/mathew-mclaren(0c35bb7e-c8f5-4a77-80fa-32cd1d456221).html)^7^, Philine Stötzner ^8^, Stefan Förster^8^, Eglantina Benavente^9,10^, Caterina Salgado^9^, Sindy Devis^1^, Paula Solar^1^, Luis Velasquez^1^, and Guillermo González^3*^.

^1^Universidad SEK, Instituto de investigación Interdisciplinar en Ciencias Biomédicas SEK (I3CBSEK), Facultad Ciencias de la Salud, Fernando Manterola 0789, Providencia, Santiago, Chile.

^2^Max Planck Institute of Microstructure Physics, Weinberg 2, D 06120, Halle, Germany.

^3^Universidad de Chile, Facultad de Ciencias, Las Palmeras 3425, Nuñoa, Santiago, Chile.

^4^Queen’s University Belfast, University Rd, Belfast BT7 1NN, United Kingdom

^5^SuperSTEM Laboratory, STFC Daresbury Campus, Daresbury WA4 4AD, United Kingdom

^6^School of Chemical and Process Engineering, University of Leeds, Leeds LS2 9JT, United Kingdom

^7^University of Exeter, Living Systems Institute, Exeter EX4 4QD, United Kingdom

^8^Martin-Luther-Universität Halle-Wittenberg, Halle, Germany**.**

^9^Departamento de Química, Facultad de Ciencias Naturales, Matemática y Medio Ambiente,

Universidad Tecnológica Metropolitana, Santiago, Chile.

^10^Programa Institucional de Fomento a la Investigación, Desarrollo e Innovación (PIDi),

Universidad Tecnológica Metropolitana, Santiago, Chile

*Corresponding authors (emails: [mmoreno@mpi-halle.mpg.de](mailto:mmoreno@mpi-halle.mpg.de), ggonzale@uchile.cl)

Scheme I shows the qualitative diagram of the positions of the semiconductor band in the TiO_2_ /ZnO heterojunction (Cheng 2014, Hernández 2014) Light radiation encompasses a vector flow of photogenerated charges that minimizes charge recombination and simultaneously prevents the accumulation of holes in the ZnO valence band that promotes ZnO degradation (Rudd 2000).

Sheme S1: ZnO + 2h^+^ +nH_2_O → Zn(OH)_n_ ^(2-n)^ + ½ O2 + nH^+^

1. **Sample T1; H_2_Ti_3_O_7_ NTs.**

Figure S1. T1 EDX.


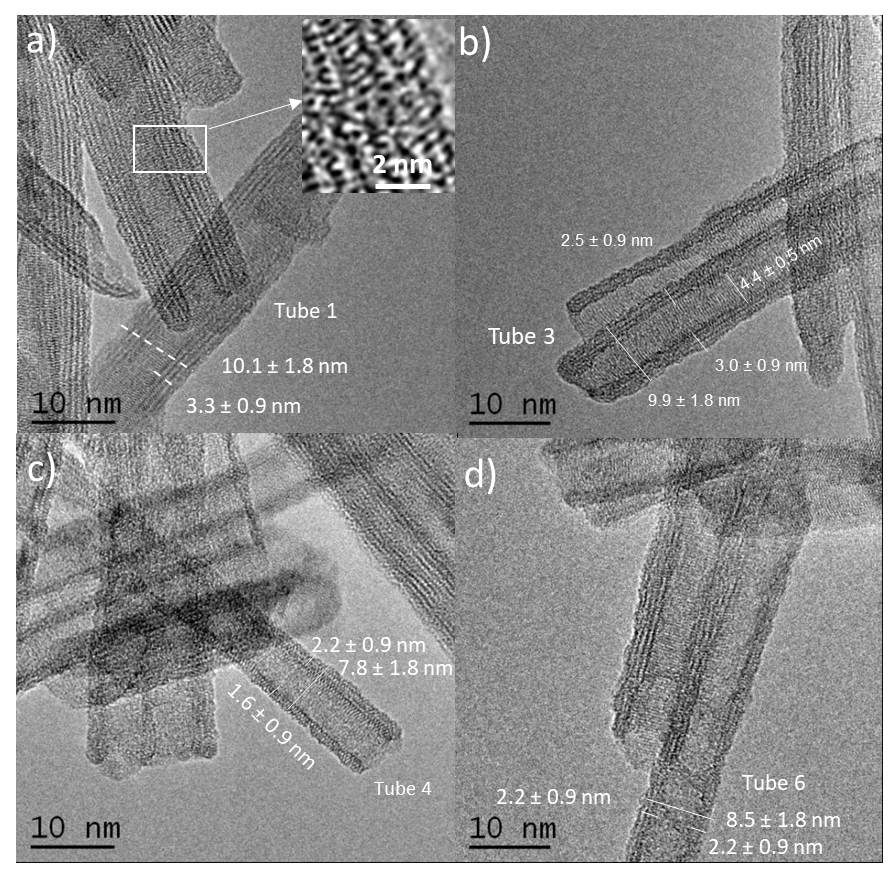


Figure S2. T1 TEM micrographs.

Table S1. The framework structure of D_2_Ti_3_O_7_ from 47-0561 JCPS (To replace 41-192). (Feist 1992)

| *(hkl)* | H_2_Ti_3_O_7_ (in 2θ)  (47-0561 JCPDS) | T1 (2θ) |
| --- | --- | --- |
| 001 | 9.82 | 9.47 ± 0.13 |
| 200 | 11.24 | 10.16 ± 0.10 |
| 201 | 13.40 |  |
| 201 | 16.39 |  |
| 002 | 19.68 | 19.37 ± 0.11 |
| 202 | 20.70 |  |
| 400 | 22.63 |  |
| 110 | 24.39 | 24.08 ± 0.13 |
| 202 | 24.66 |  |
| 111 | 25.98 |  |
| 401 | 26.45 |  |
| 402 | 26.94 | 28.54 ± 0.63 |
| 310 | 29.28 |  |
| 003 | 29.78 | 28.54 ± 0.63 |
| 311 | 32.08 |  |
| 312 | 33.56 |  |
| 203 | 33.96 |  |
| 602 | 36.07 |  |
| 601 | 37.64 |  |
| 113 | 37.96 |  |
| 113 | 39.71 |  |
| 004 | 40.07 | 39.91 ± 0.55 |
| 603 | 41.03 |  |
| 204 | 43.95 |  |
| 711 | 46.57 |  |
| 114 | 48.42 | 47.45 ± 0.25 |


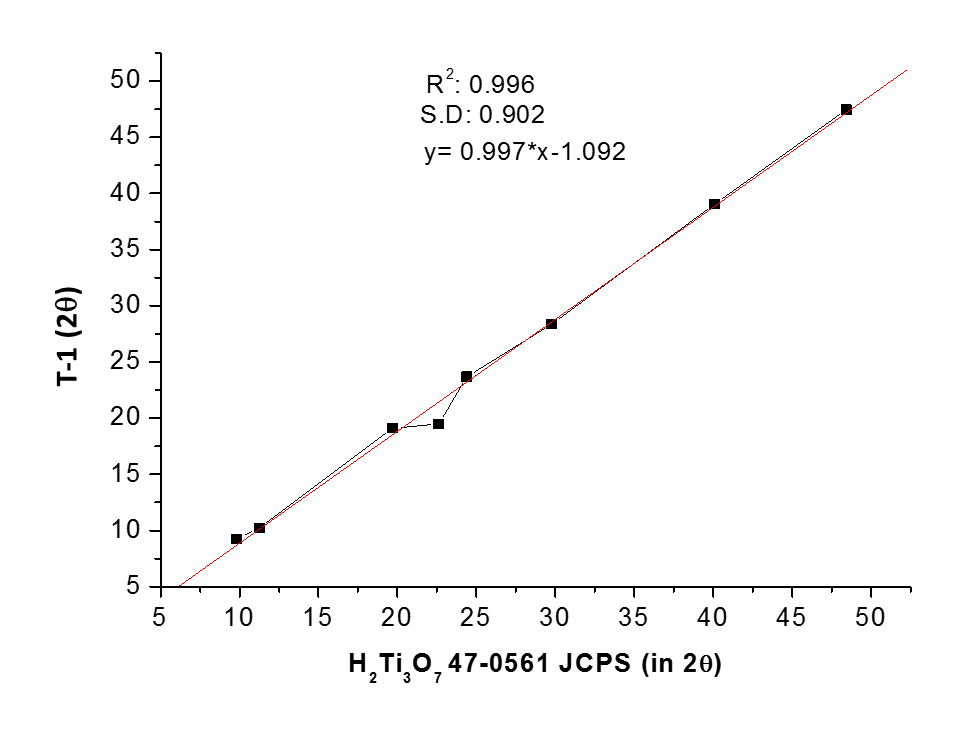


Figure S3. Linear correlation between T1 and H_2_Ti_3_O_7_ XRD patters 47-0561 JCPS. (Feist 1992) Results of linear correlation between the main T1 XRD peaks with TiO_2_(B) patterns of 0.999 (slope), 0.994 (correlation factor (R^2^)), and 0.194 (standard deviation (S.D)), revels their good agreement. Thus, the reflection peaks at 9.14° (9.67Å) and 10.18° (8.8Å) in 2θ close to 9.81° (9.1 Å) and 11.24° (~8 Å), can be indexed tentatively to *(001)* and *(200)* Miller indices of H_2_Ti_3_O_7_ with crystal lattice parameters of a: 16.02, b:3.75 and c: 9.20Å (JCPDS 47- 0561) and β = 101.45°, allowing to design to the T-1 to H_2_Ti_3_O_7_ NTs, but with general form of H_2_Ti_3_O_7_·3.14H_2_O.


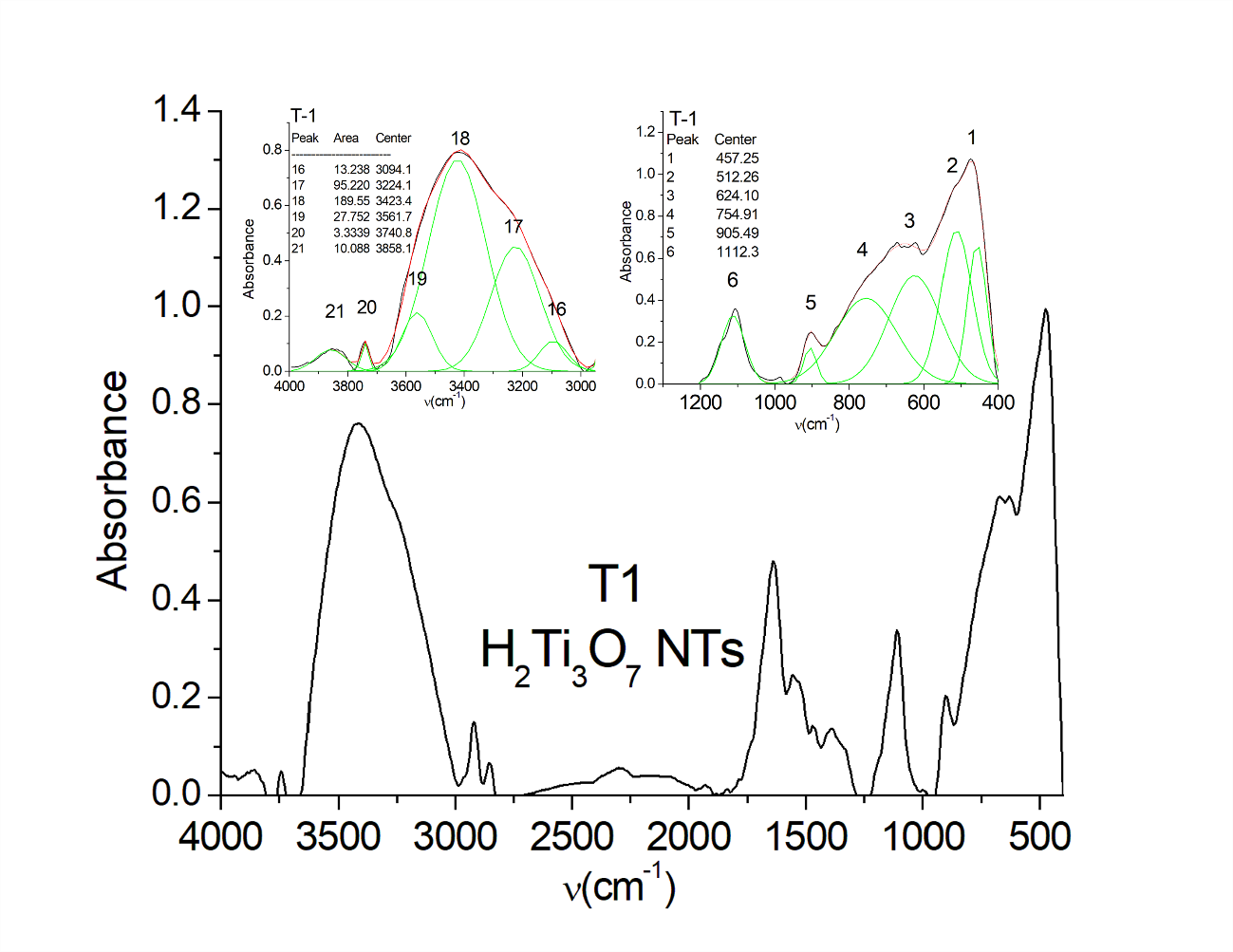


Figure S4. T1 IR spectrum in the range of 450-4000 cm^-1^. Deconvoluted IR bands in the range of 450-1250 cm^-1^ (right inset) and 3000-4000 cm^-1^(left inset).


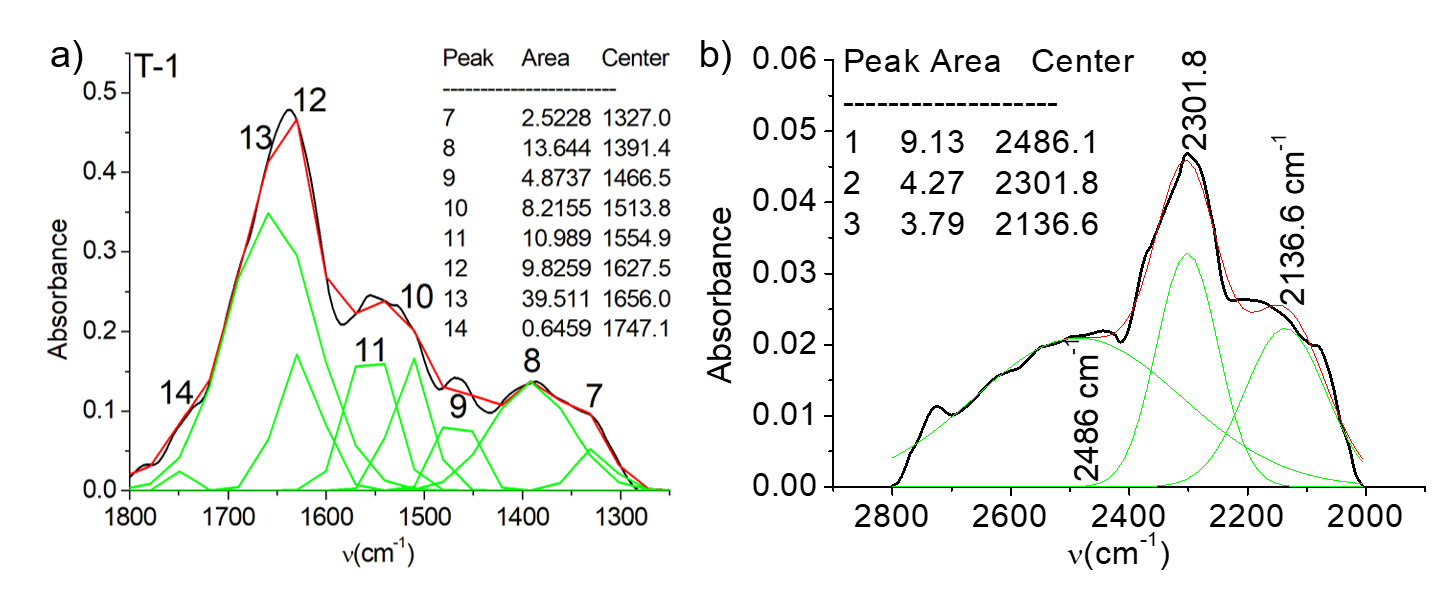


Figure S5. Deconvoluted T1 IR bands in the range of 1250-1800 cm^-1^ (a), and 2000-2800 cm^-1^(b), performing a Gaussian function (Bradley M., 2007) in Origin 6.0.





Figure S6. T1 IR Linear correlation between calculated (using Falk´s equation) and experimental νOH.

Table S2. Bend (libration “ν_L_” and scissor “ν_2_”) and stretch (νOH) of water IR bands in T1, assignments based in overtones and bands combinations. Where ν_L_ is rocking “ρ”, twisting “τ”, wagging “ω”, ν_2_ is scissor “δ” and νOH is average between ν_1_ and ν_3_.

| Exp. T1 in cm^-1^ | | Overtones | | Bands combinations | |
| --- | --- | --- | --- | --- | --- |
| νL (Petruševski V, 1990) | | 2ν | 3ν |  |  |
| 473.7 ± 3.4 | νL1 ρ(H_2_O) | 947.5 | 1421.2 | n.d | n.d |
| 550.3 ± 7.5 | νL2 ρ(H_2_O) | 1100.6 | 1651.0 | n.d | n.d |
| 670.3 ± 0.4 | νL3 ρ(H_2_O) | 1340.6 | 2010.9 | n.d | n.d |
| 750.0 ± 9.9 | νL4 τ(H_2_O) | 1500.0 | 2250.1 | n.d | n.d |
| 909.0 ± 4.1 | νL5 ω(H_2_O) | 1818.1 | 2727.1 | 2νL1 | 947.5 |
| ν2 (scissor “δ”) (Vinaykin M, 2012) | |  |  |  | |
| 1113.8 ± 3.7 | ν2.0 δ(H_2_O) | 2227.6 | 3341.4 | 2νL2 | 1100.6 |
| 1332.5 ± 5.3 | ν2.1 δ(H_2_O) | 2665.0 | 3997.4 | 2νL3 | 1340.5 |
| 1396.4 ± 4.1 | ν2.2 δ(H_2_O) | 2792.8 | 4189.2 | n.d | n.d |
| 1464.3 ± 5.1 | ν2.3 δ(H_2_O) | 2928.5 | 4392.7 | n.d | n.d |
| 1515.7 ± 6.8 | ν2.4 δ(H_2_O) | 3031.4 | 4547.1 | 2νL4 | 1500.0 |
| 1559.6 ± 2.0 | ν2.5 δ(H_2_O) | 3119.1 | 4678.7 | n.d | n.d |
| 1640.4 ± 7.0 | ν2.6 δ(H_2_O) | 3280.7 | 4921.1 | n.d | n.d |
| 1659.4 ± 3.3 | ν2.7 δ(H_2_O) | 3318.9 | 4978.3 | 3νL2 | 1650.9 |
| 1696.6 ± 3.3 | ν2.8 δ(H_2_O) | 3393.2 | 5089.8 | n.d | n.d |
| 1738.3 ± 3.8 | ν2.9 δ(H_2_O) | 3476.7 | 5215.0 | n.d | n.d |
| 1763.1 ± 4.4 | ν2.10 δ(H_2_O) | 3526.3 | 5289.4 | n.d | n.d |
| Assignments based on bands combinations | | | | | |
| 2136.6 ±2.1 | νL1+νL2+ ν2.0 | 2137.9 | νL1+3νL2 | 2124.7 | |
| 2301.8 ±3.4 | 5νL1  3νL4 | 2368.7  2250.0 | νas CO_2_ | 2340.0 | |
| 2486.1 ± 1.1 | νL5+ν2.5 | 2468.6 | 2νL5+νL3 | 2488.4 | |
| 2723.0 ± 2.7 | 3νL5 | 2727.1 | 2ν2.2 | 2792.8 | |
| 2853.8 ± 0.69 | ν2.0+ν2.9 | 2851.8 | 2(3νL1) | 2842.4 | |
| 2919.1 ±2.4 | 2ν2.3 | 2928.5 | 5νL1+νL2 | 2919.0 | |
| 2966.4 ±6.4 | 3νL4+νL3 | 2920.4 | νL1+νL3+2νL5 | 2962.1 | |
| 3094.1 ± 4.4 | 2ν2.5 | 3898.9 | 4νL4 | 3000.1 | |
| 3224.1 ± 3.7 | 2ν2.6 | 3280.6 | 7νL2 | 3852.2 | |
| 3429.06±7.7 | 3νL4+νL3+νL1 | 3394.1 | 5νL2+νL3 | 3421.9 | |
| 3561.7 ± 3.3 | 6νL1+νL4 | 3592.4 | 2ν2.10 | 3526.2 | |
| 3740.0 ± 5.5 | 4νL4+νL3 | 3670.4 | 8νL1 | 3789.8 | |
| 3858.1± 1.7 | νL5+2ν2.3 | 3837.8 | 7νL1+νL2 | 3866.4 | |
| 3936.0 ± 1.3 | 2ν2.8+νL2 | 3943.5 | 2ν2.9+νL1 | 3950.4 | |

1. **Sample T2; (ZnO)Ti_3_O_7_NTs by VPM.**


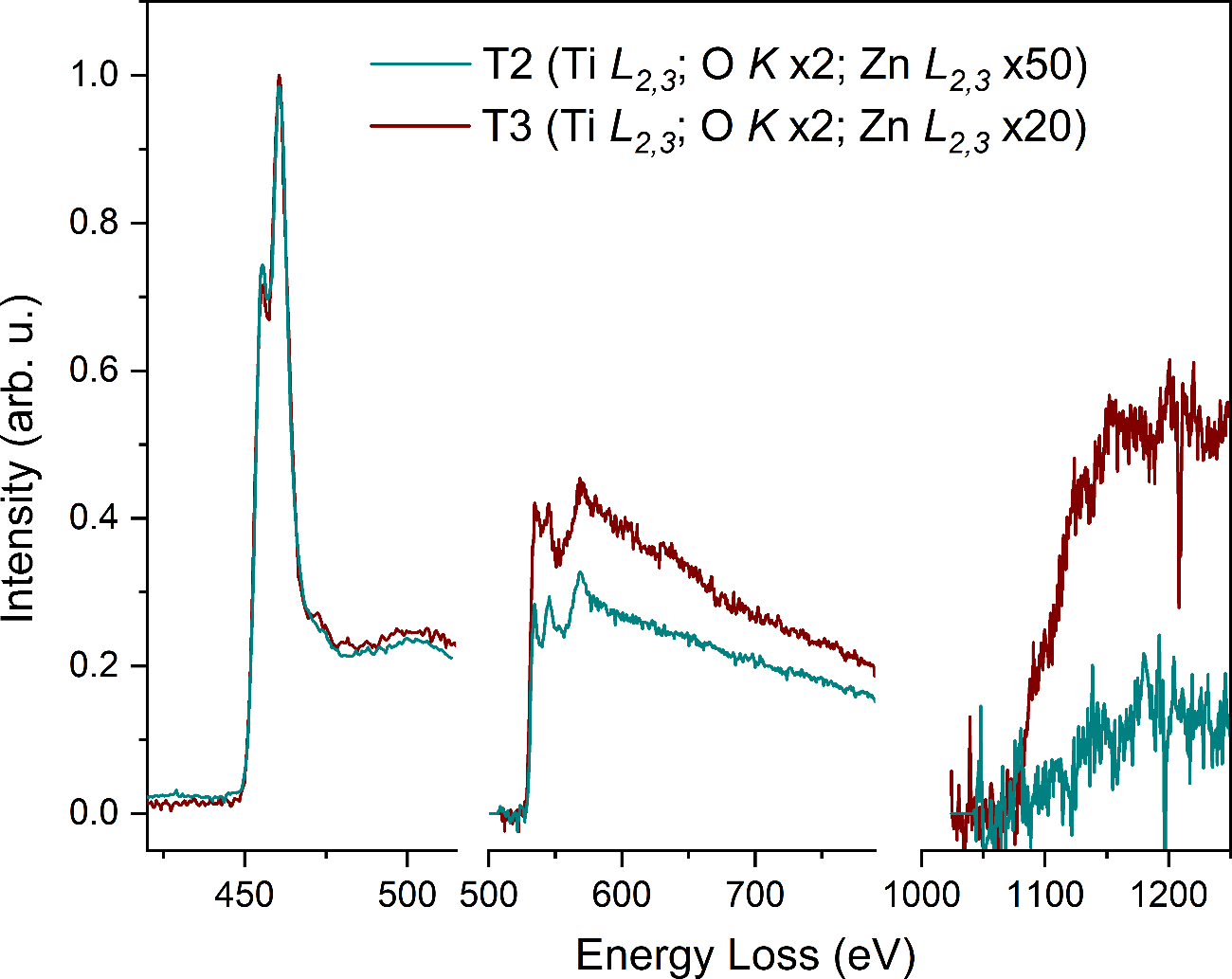


Figure S7. Comparison of the relative shape and intensity of electron energy loss spectra averaged over representative tubes from the T2 and T3 samples and normalized to the Ti L_2_ peak maximum.

**
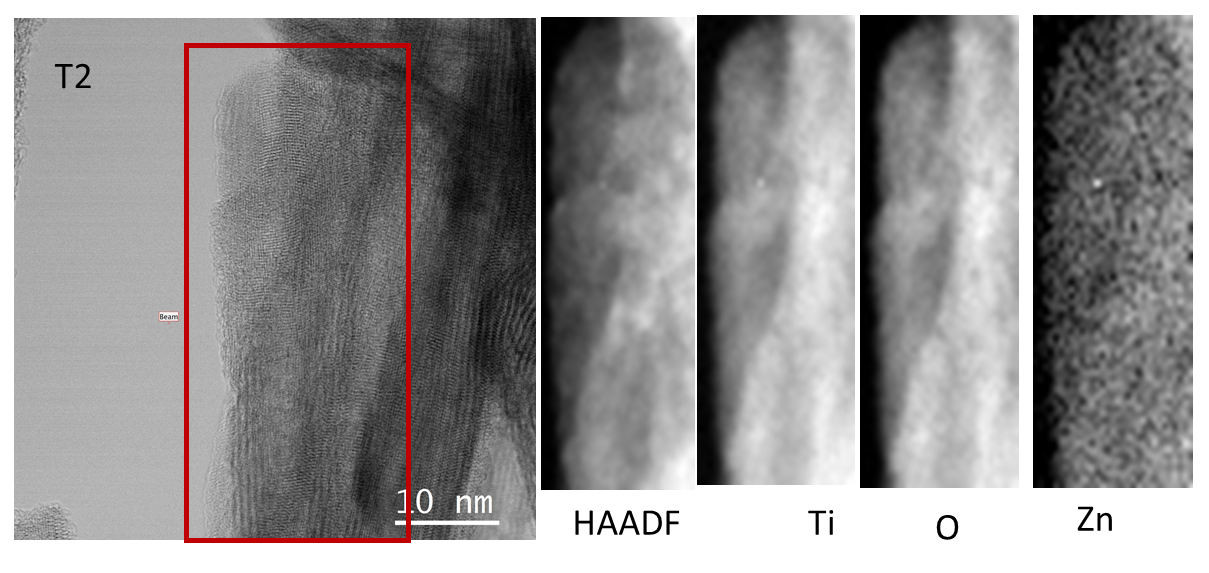
**

Figure S8. STEM micrography and Electron energy loss spectral (EELS) of T2. From left to right, bright field (BF – obtained prior to carrying out the EELS acquisition), high-angle annular dark-field (HAADF); Ti-L_2,3_, O-K and Zn-L_2,3_ elemental maps.


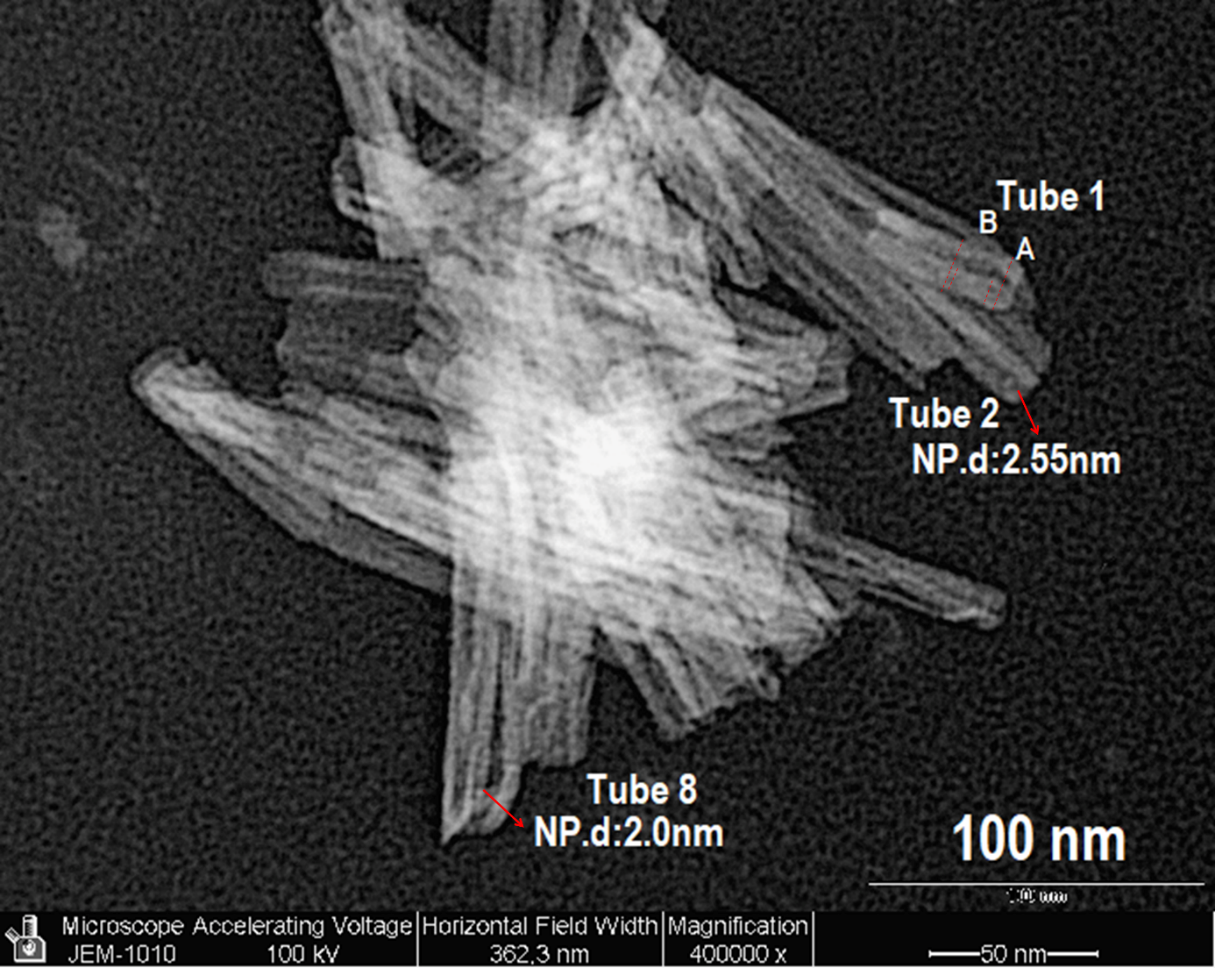


Figure S9. T2 micrograph, showing nanoparticles and widening of the NTs from 1 to 8. For instance, NTs 1, 2 and 8 reveal NPs of ~2.30 nm. And NT 1 depicts the increment of wall (W) and the total diameter (T.D) from 8.26 ± 1.62 nm and 16.15 ± 2.35 nm in B to 8.86 ± 1.62 nm and 17.10 ± 2.35 nm in A. The micrograph’s contrast has been adjusted to emphasize thin tubes, resulting in thicker areas appearing over-exposed.


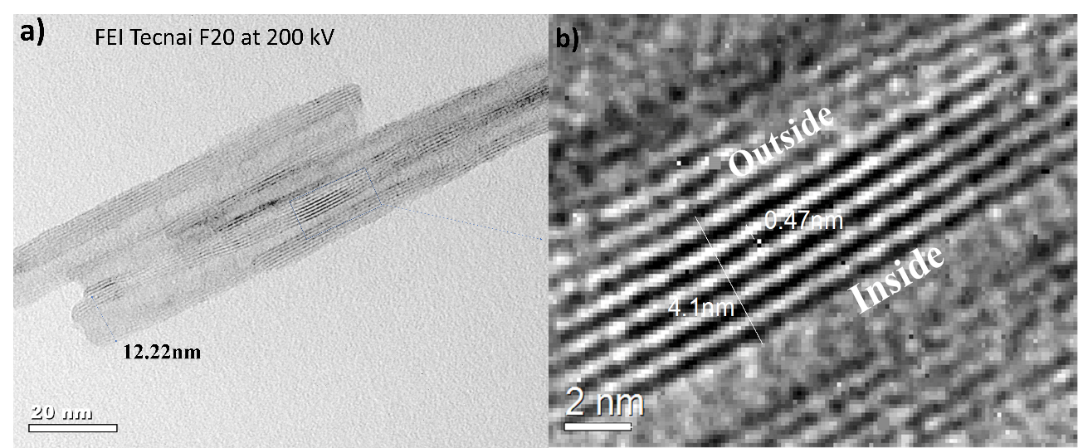


Figure S10. T2 micrograph.


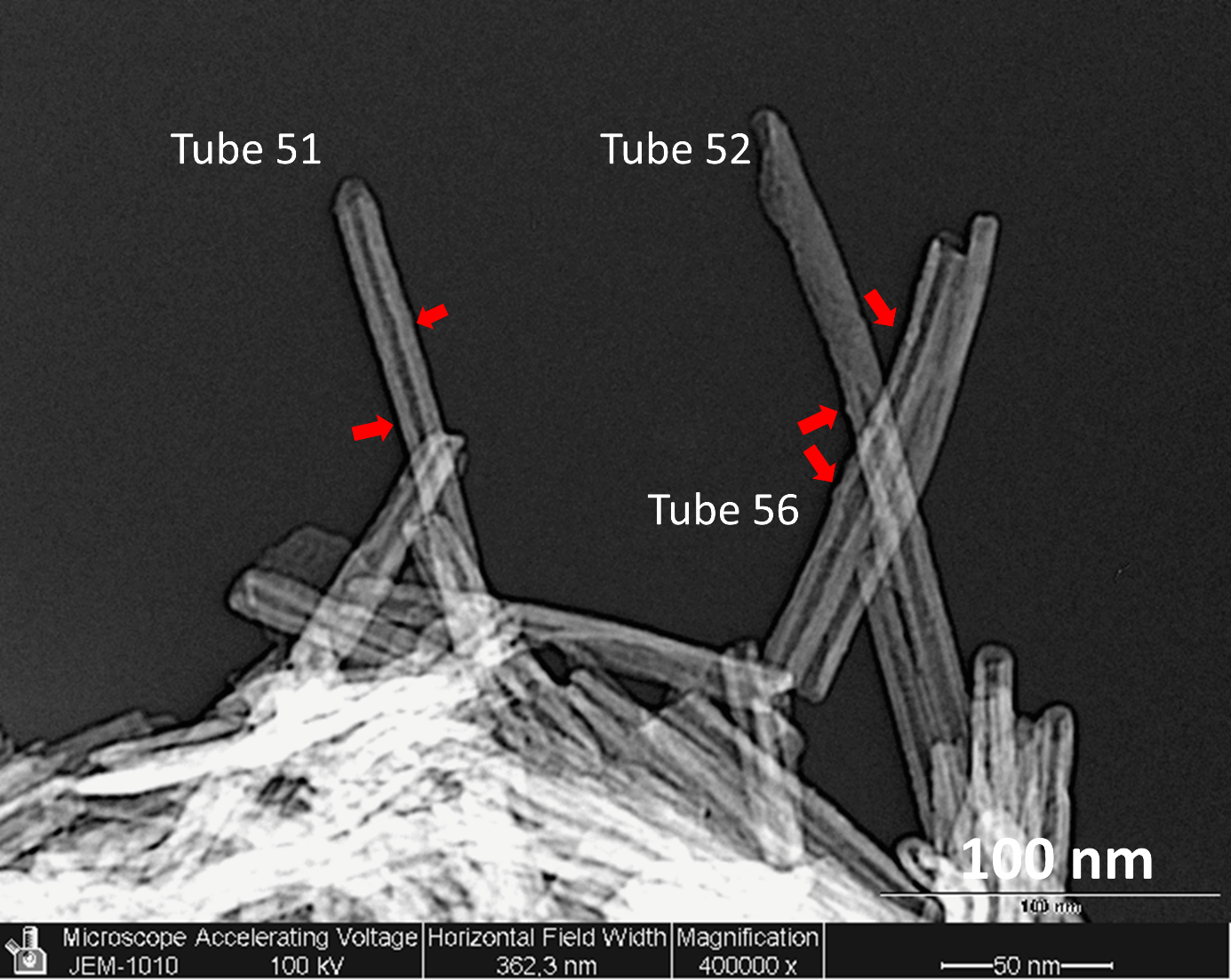


Figure S11. T2 micrograph, showing widening, small deformations, and fringes in different regions of the NTs from 51 to 56 (red arrows). The micrograph’s contrast has been adjusted to emphasize thin tubes, resulting in thicker areas appearing over-exposed.


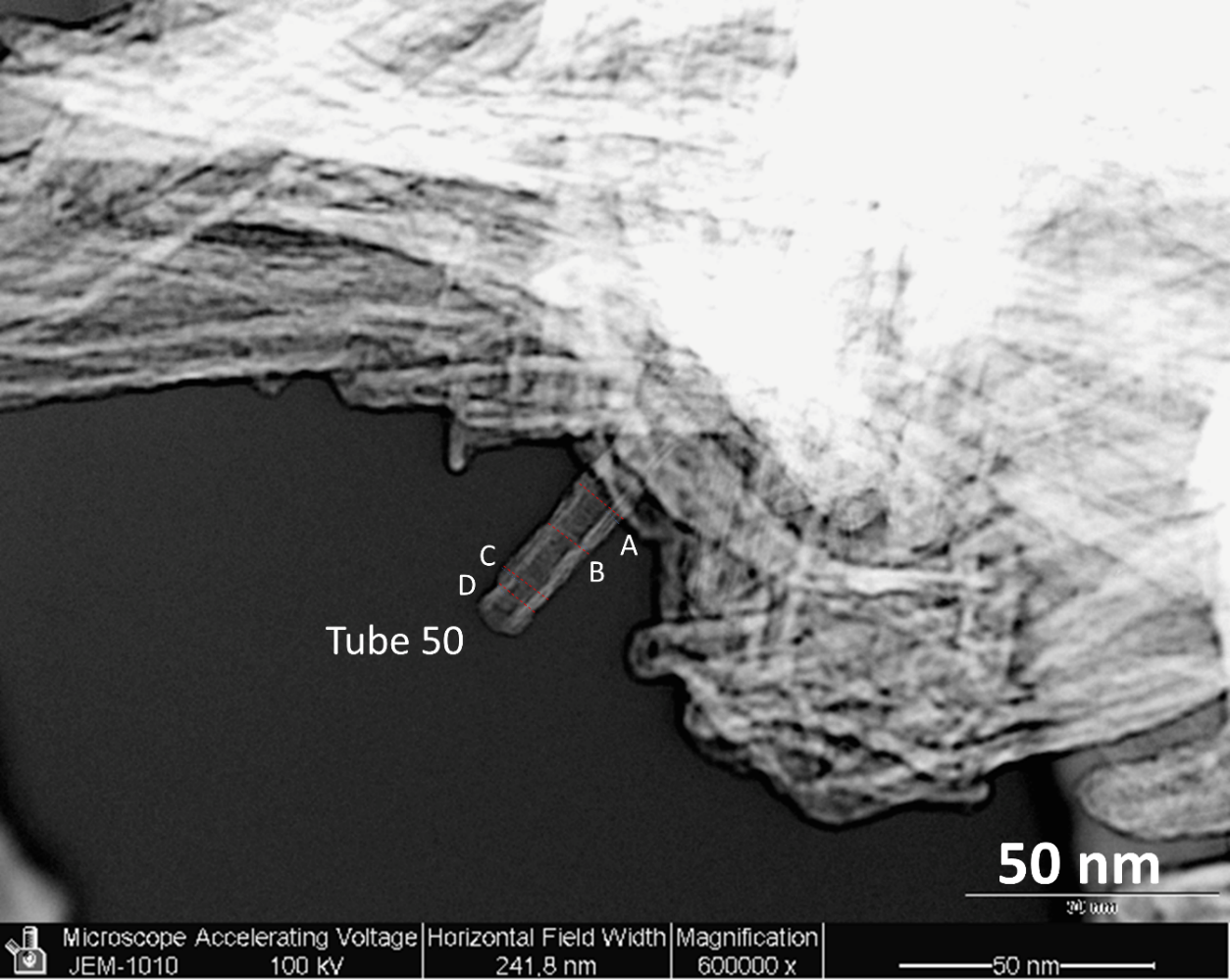


Figure S12. T2 micrograph, showing widening and deformations in different regions of NTs from 48 to 50. For instance, NT 50 with wall and total diameter of 3.60 ± 1.62 nm and 12.07 ± 2.35 nm in A, 2.37 ± 1.62 nm and 10.28 ± 2.35 nm in B, 4.31 ± 1.62 nm and 11.54 ± 2.35 nm in C, and 2.24 ± 1.62 nm and 9.05 ± 2.35 nm in D.


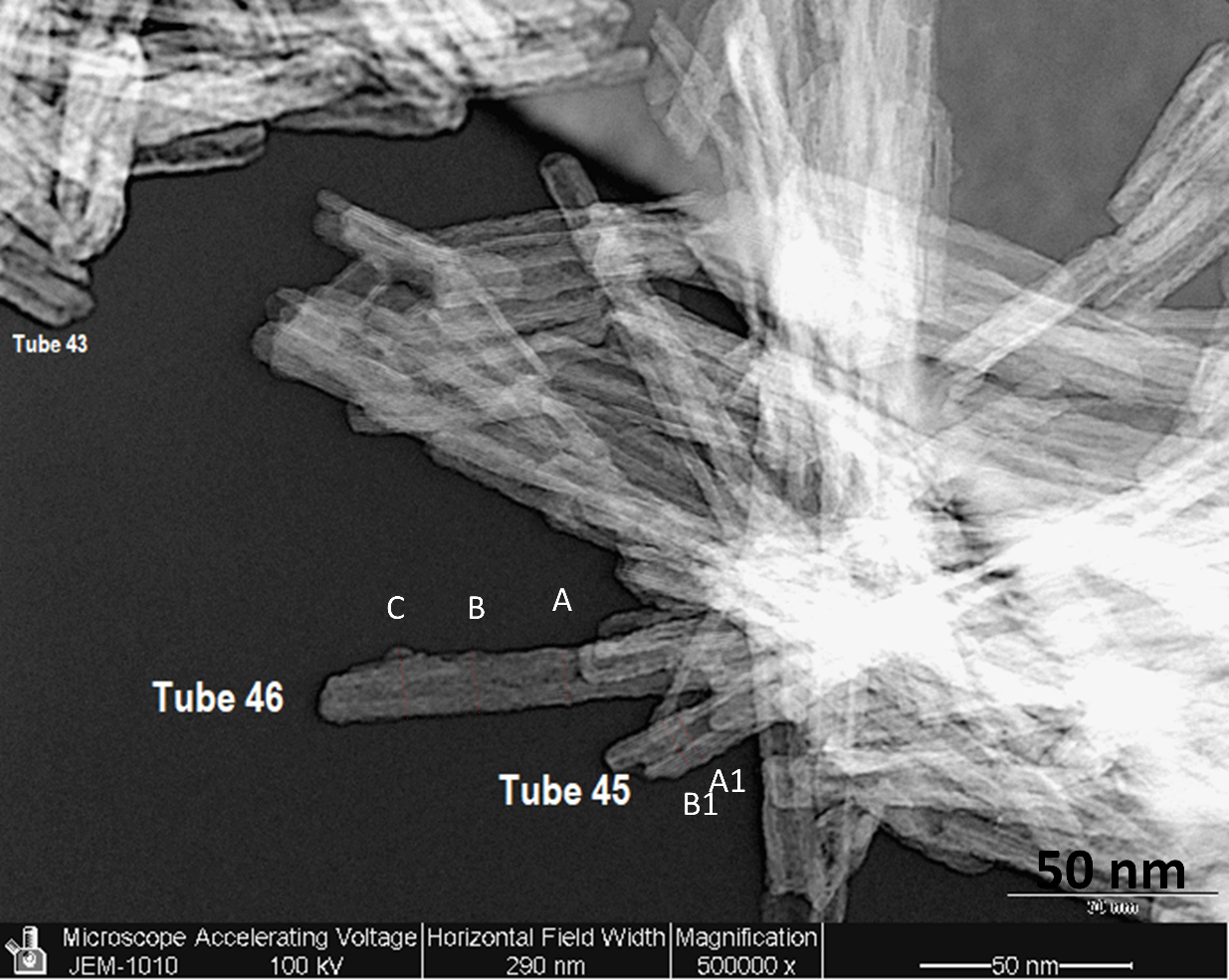


Figure S13. T2 micrograph, showing widening, deformations, and fringes in different regions of the NTs from 42 to 46. For instance, NT 45 reveals an increasing of wall (W) and the total diameter (T.d) from 5.04 nm and 13.56 ± 2.35 nm in B to 6.40 nm and 14.64 ± 2.35 nm in A, respectively. Tube 46 depicts a consecutive increment of T.d from A of 13.86 ± 2.35 nm, passing for B of 15.53 ± 2.35 nm to C of 17.70 ± 2.35 nm, respectively. The region between the high contrasted fringes (C and A1), where small particles are apparent, points to the replacement of water molecules in the original nanotubes by intercalated zinc species. The micrograph’s contrast has been adjusted to emphasize thin tubes, resulting in thicker areas appearing over-exposed.


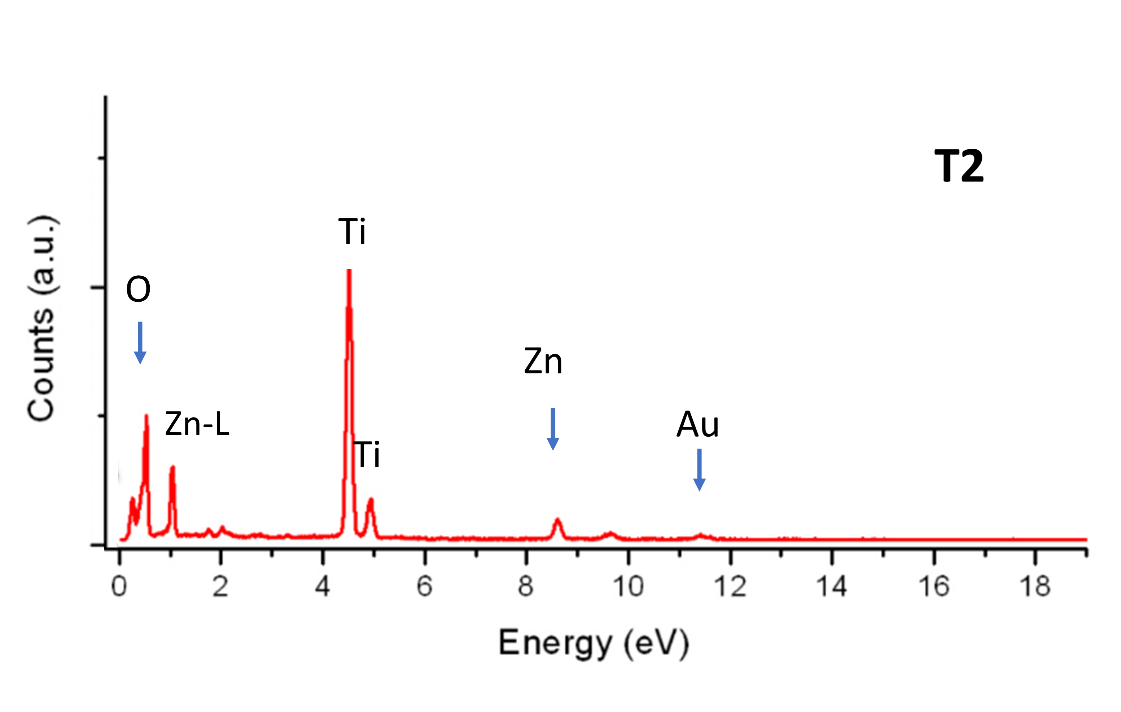


Figure S14. T2 EDX.





Figure S15. It shows abundant nucleated ZnO NPs in Ti_3_O_7_ NTs obtained setting the ALD chamber at RT instead of 120°C.


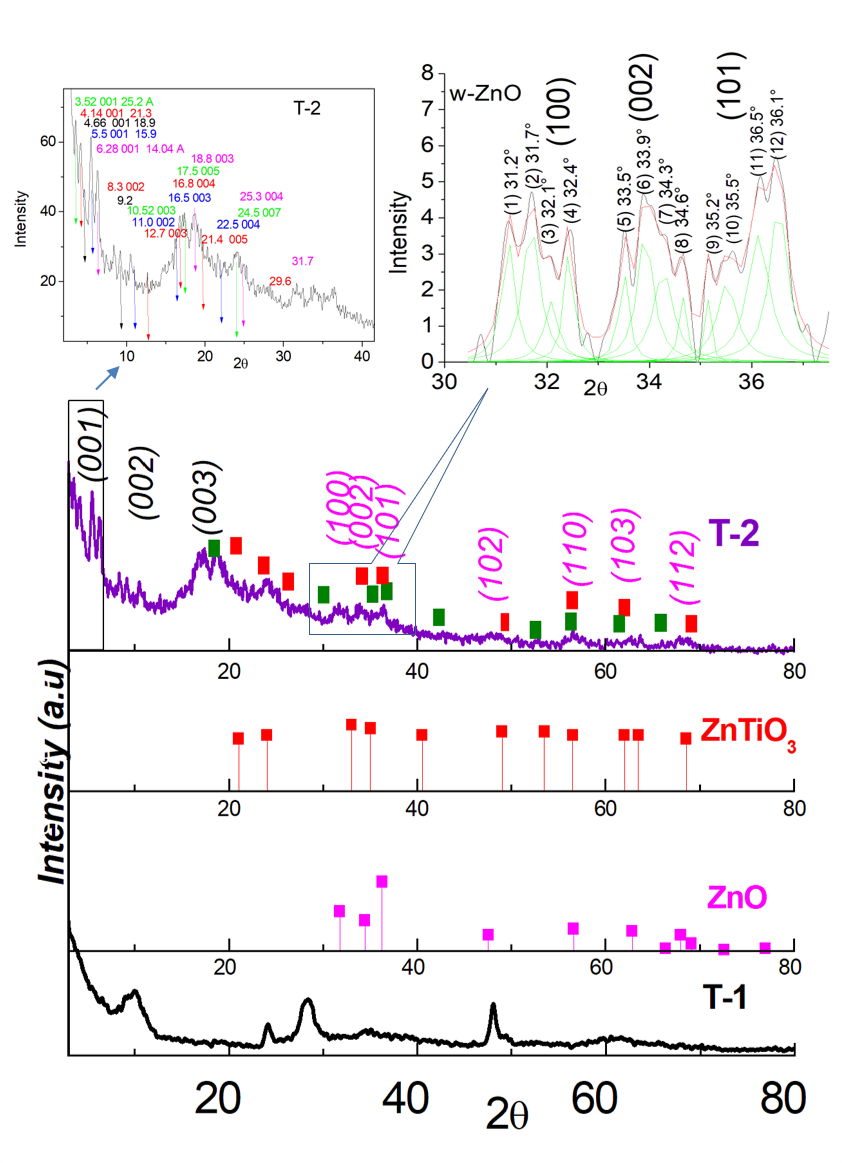


Figure S16. XRD of T2 in range 2.5-80° in 2θ; T2 (Violet), w-ZnO (magenta) ICDD card 361451, ZnTiO_3_ (red) ICDD 26-150, and T- 1 (black), in range 2.5-5° in 2θ, showing *(00l)* family of planes (left-inset), and in range 30-38° in 2θ (right-inset).

Table S3. T2 and T3 XRD planes (*00l*) at low and high angle, and distances in Å using Bragg´s law.

| 2θ (001) | $d_{(001)}$* | 2θ (002) | $d_{(002)}$ | | 2θ (003) | | 2θ (004) | | 2θ (005) | | 2θ  (006) |
| --- | --- | --- | --- | --- | --- | --- | --- | --- | --- | --- | --- |
| Low angle analysis of T2 | | | | | | | | | | | |
| 3.61 ± 0.09 | 24.45 | 7.22 | 12.23 | | 10.83 | | 14.44 | | 18.05 | | 21.66 |
| 4.25 ± 0.11 | 20.96 | 8.42 | 10.49 | | 12.63 | | 16.84 | | 21.05 | | 25.26 |
| 5.56 ± 0.12 | 16.05 | 11.00 | 8.03 | | 16.50 | | 22.00 | | 27.50 | | 33.00 |
| 6.36 ± 0.08 | 14.06 | 12.56 | 7.04 | | 18.84 | | 25.12 | | 31.40 | | 37.68 |
| High angle analysis of T2, presence of five types of w-ZnO nanocrystals confined in *d_(001)_.* | | | | | | | | | | | |
|  | 2θ *(100)* | $d_{(100)}$ | a_0_^a^ |  | 2θ *(002)* | $d_{(002)}$ | $d_{(001)}$ | c_0_^a^ |  | 2θ (101) | d(101) |
| 31.62±0.72 | 30.7 | 2.91 | 3.2Å | 33.82±0.72 | 32.78 | 2.73 | 5.45 | 5.2Å | 36.07±0.75 | 35.2 | 2.55 |
|  | 31.2 | 2.86 |  |  | 33.50 | 2.67 | 5.34 |  |  | 35.5 | 2.53 |
|  | 31.7 | 2.82 |  |  | 33.90 | 2.64 | 5.28 |  |  | 36.5 | 2.46 |
|  | 32.1 | 2.79 |  |  | 34.30 | 2.61 | 5.22 |  |  | 36.1 | 2.49 |
|  | 32.4 | 2.76 |  |  | 34.60 | 2.59 | 5.18 |  |  | 37.07 | 2.42 |
|  | | | | | | | | | | | |
| T3 XRD planes (00l) at low angle. | | | | | | | | | | | |
| 2θ (001) | $d_{(001)}$* | 2θ (002) | | $d_{(002)}$ | | 2θ (003) | | 2θ (004) | | 2θ (005) | |
| 4.32±0.04 | 20.43 | 8.64 | | 10.22 | | 12.96 | | 17.28 | | 21.60 | |
| 7.05±0.03 | 12.52 | 14.10 | | 6.27 | | 21.15 | | 28.20 | | 32.25 | |
| 8.28±0.07 | 10.67 | 16.56 | | 5.35 | | 24.84 | | 32.12 | | 41.4 | |
| 9.40±0.21 | 9.40 | 18.80 | | 4.71 | | 28.20 | | 37.60 | | 47.00 | |
| 10.19± 0.18 | 8.67 | 20.38 | | 4.35 | | 30.57 | | 40.76 | | 50.95 | |

a: corrections to obtain experimental a_0_ and c_0_ can be found in Bunn´s report. (Bunn C. W., 1935).

*****$d_{(hkl)}=\left( \frac{1.54Å}{2\sin\theta} \right)$

**
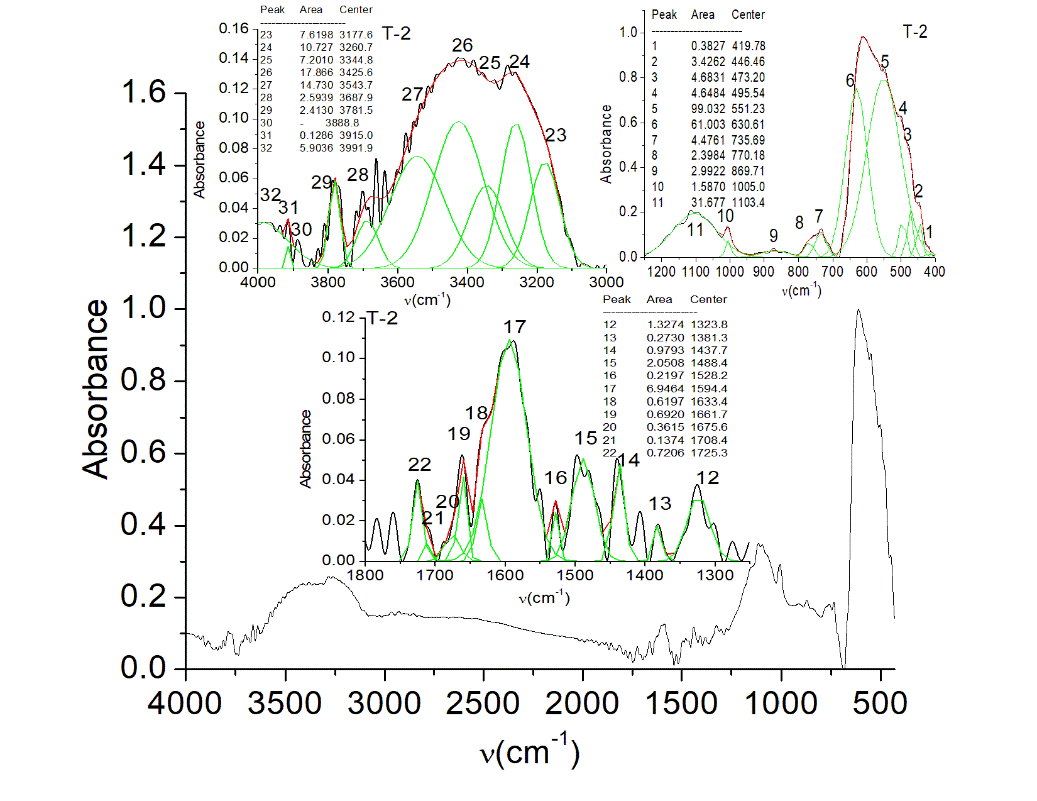
**

Figure S17. T2 IR spectrum in range of 450-4000 cm^-1^. Deconvoluted IR bands in the range of 450-1250 cm^-1^ (right inset), 3000-4000 cm^-1^(left inset) and 1000-1800 cm^-1^ (down-inset).


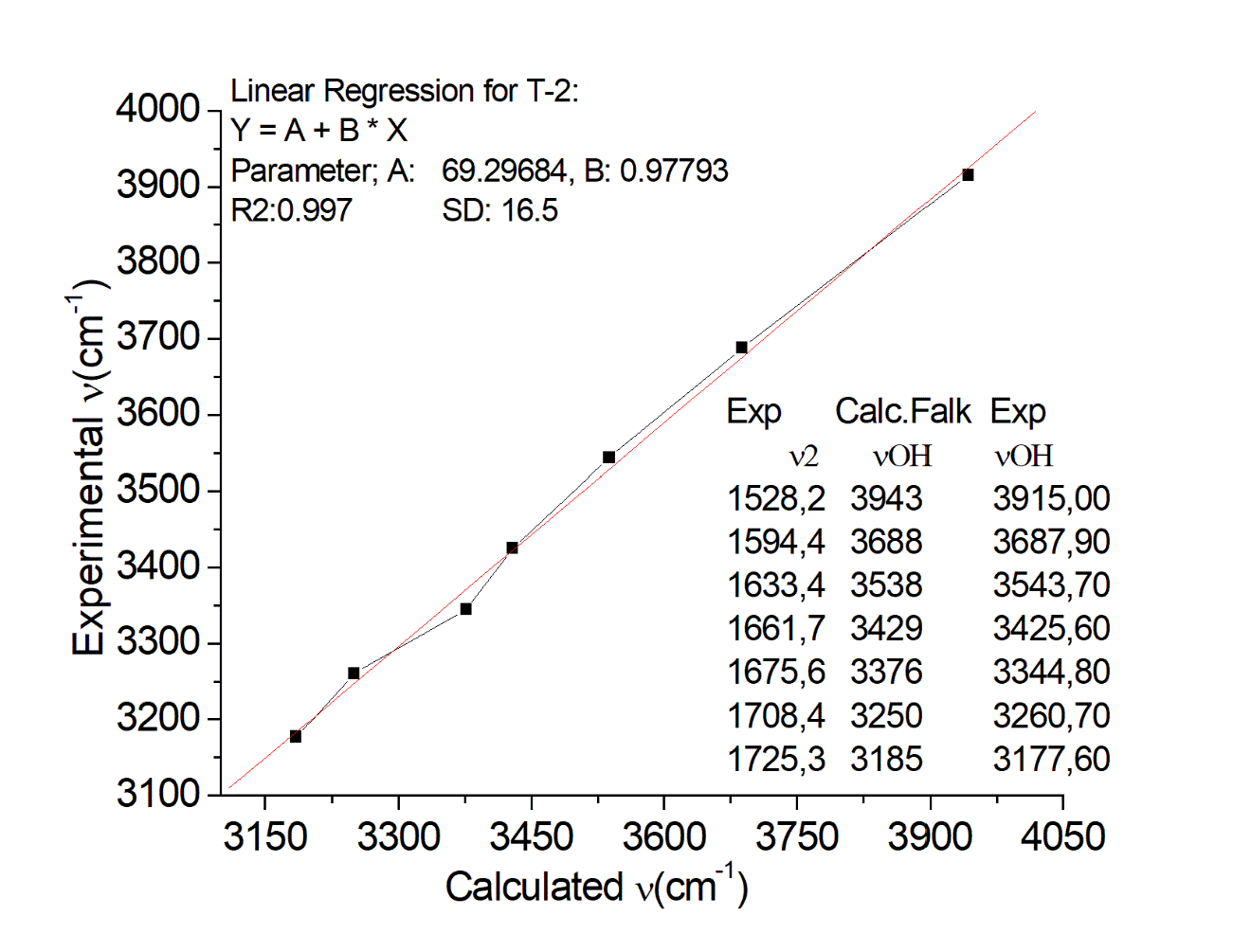


Figure S18. T2 IR linear correlation between calculated (Falk´s equation) and experimental νOH.

1. **T3: (ZnO)Ti_3_O_7_NTs/NPs by MPI.**

**
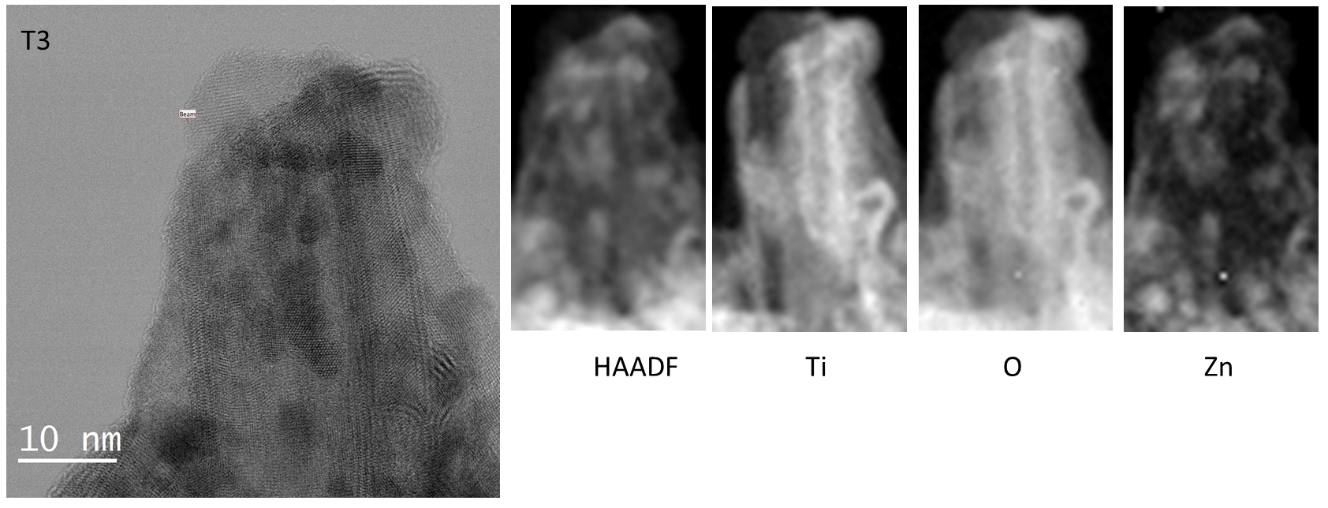
**

Figure S19. STEM micrography and Electron energy loss spectral (EELS) of T3. From left to right, bright field (BF), high-angle annular dark-field (HAADF); Ti-L_2,3_, O-K and Zn-L_2,3_ elemental maps.


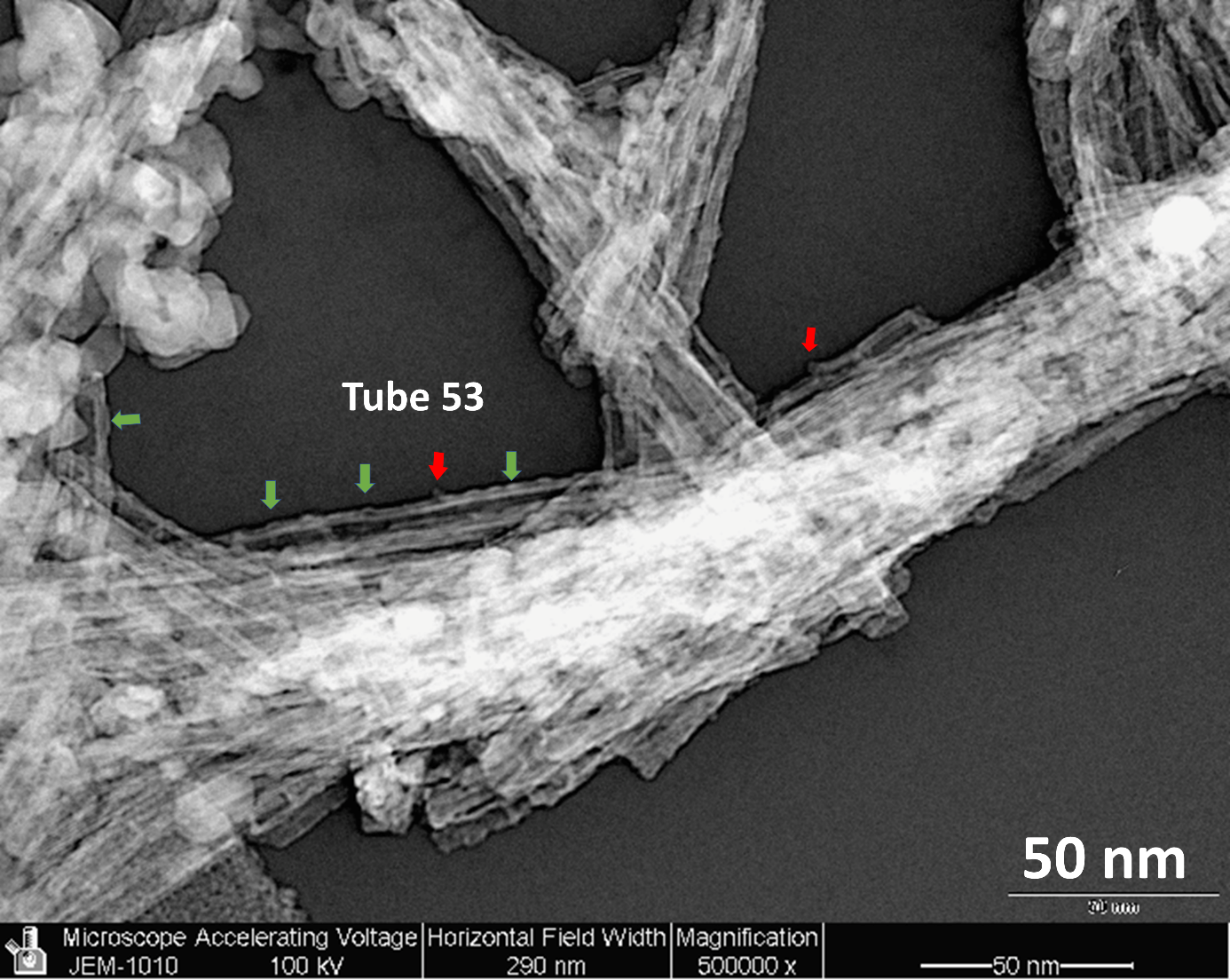


Figure S20. T3 micrograph, showing widening, deformations, and fringes (green arrows) in different regions of NT 53 and fractures (red arrows). The structure of the zone between the high contrasted fringes, where many very small particles are apparent, points to the replacement of water molecules in the original nanotubes by intercalated zinc species. The micrograph’s contrast has been adjusted to emphasize thin tubes, resulting in thicker areas appearing over-exposed.


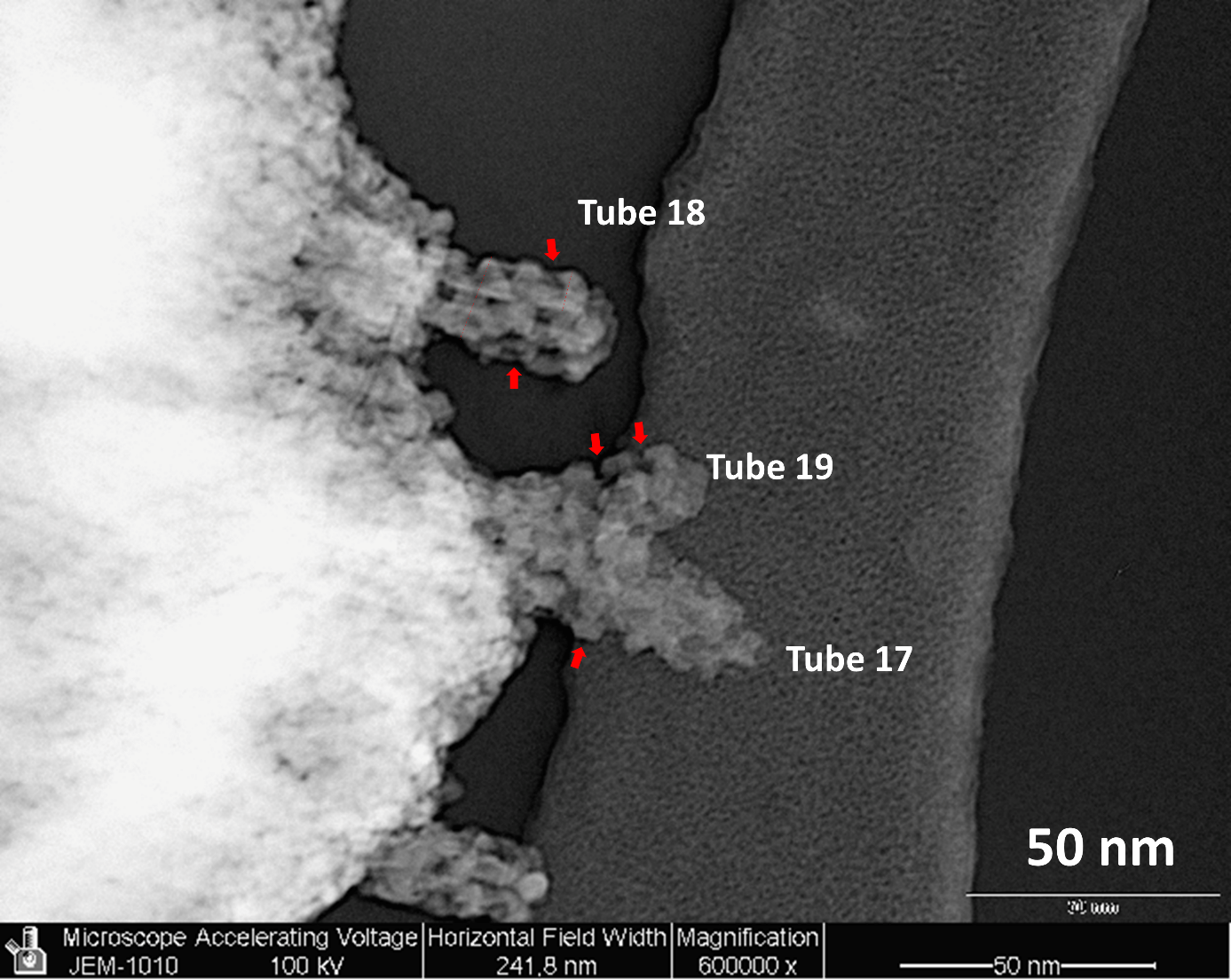


Figure S21. T3 micrograph, showing widening, deformations, fringes, demolition (red arrows) and ZnO nucleation in different regions of the NTs from 17 to 20. For instance, NT 18 shows localized demolition which could be product of the growth of ZnO nanoparticles in the channel, between the layers and on the surface.


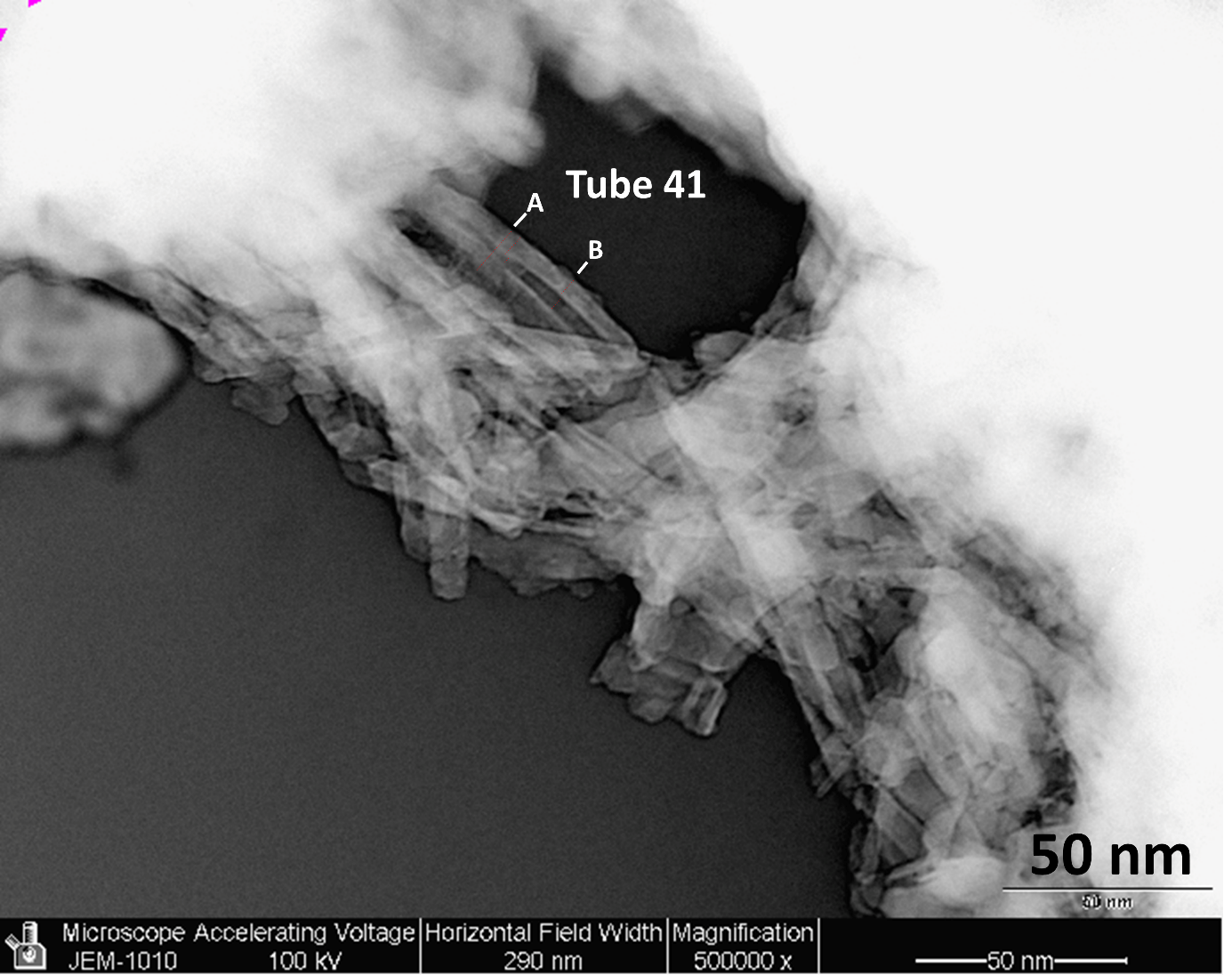


Figure S22. T3 micrograph, showing widening and decreasing in different regions of NTs 1. For instance, increment in wall and total diameter from 5.30 ± 2.30 nm and 12.50 ± 2.84 nm in B to 6.06 ± 2.30 nm and 13.56 ± 2.30 nm in A, respectively. And decrease of inner diameter (I.d) from 4.55 ± 0.81 nm in B to 2.50 ± 0.81 nm in A.


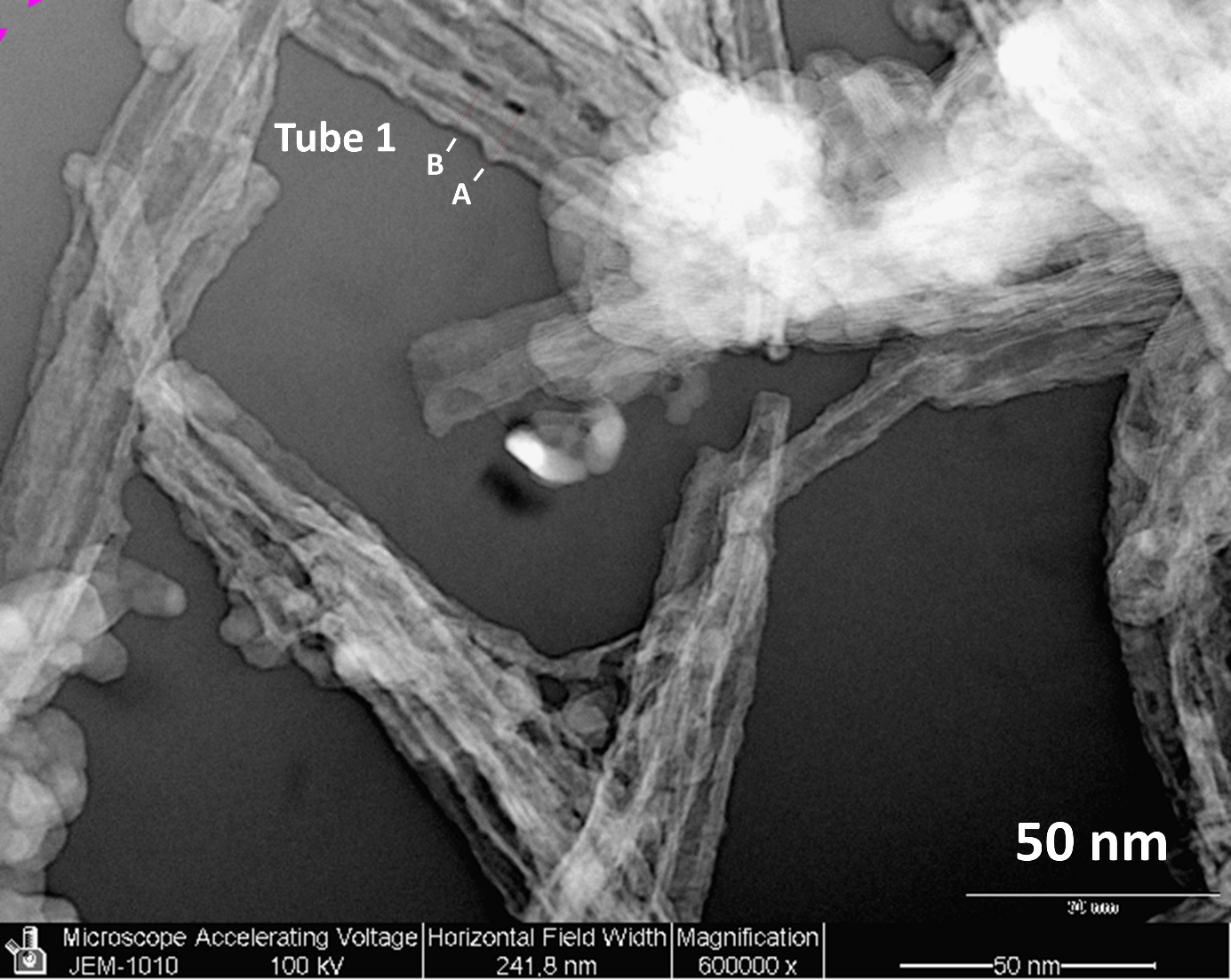


Figure S23. T3 micrograph, showing widening, deformations, and fringes in different regions of NT 1. For instance, increment in wall and total diameter from 3.22 ± 2.30 nm and 9.36 ± 2.84 nm in B to 4.44 ± 2.30 nm and 11.24 ± 2.84 nm in A. The structure of the zone between the high contrasted fringes, where many very small particles are apparent, points to the replacement of water molecules in the original nanotubes by intercalated zinc species. The micrograph’s contrast has been adjusted to emphasize thin tubes, resulting in thicker areas appearing over-exposed.

~~
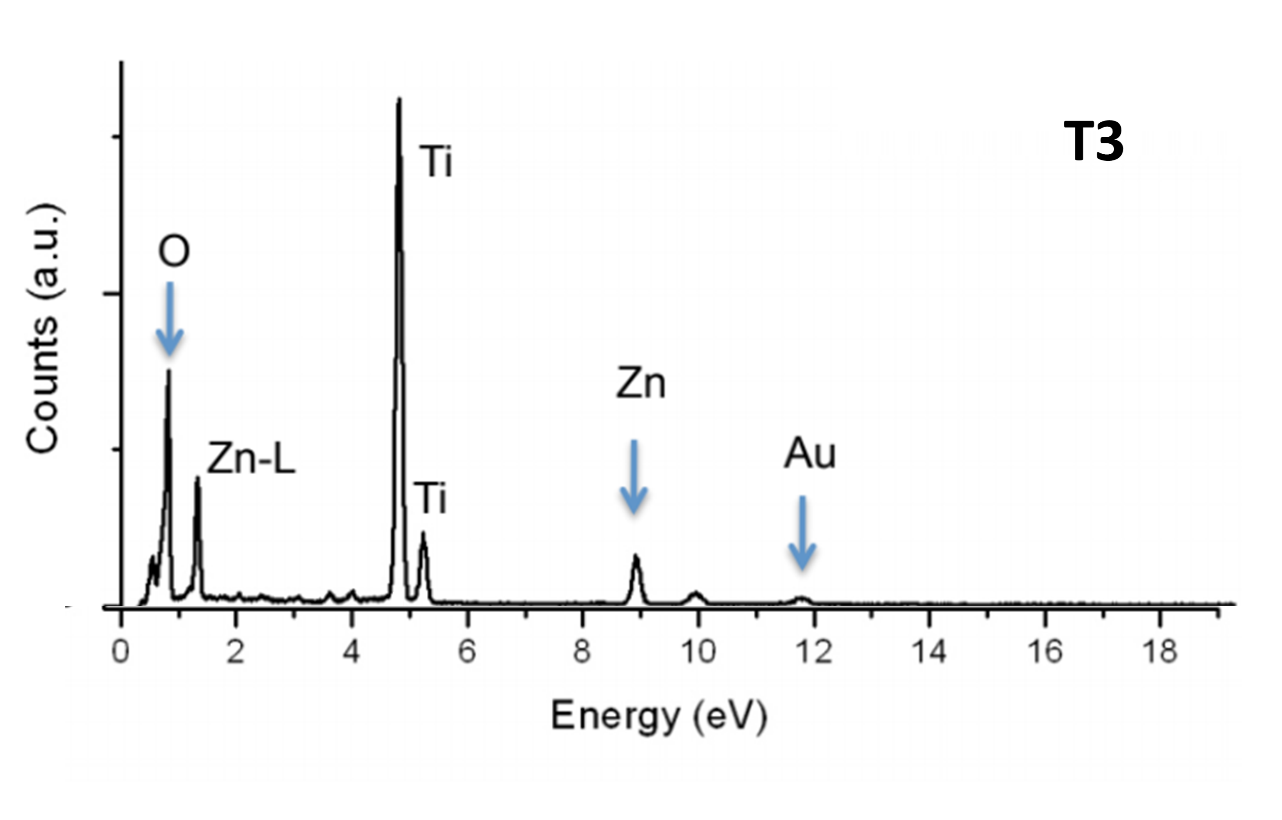
~~

Figure S24. T3 EDX.

Table S4. X-ray photoelectron spectroscopy (XPS) of T1, T2 and T3. Binding energies of O1s component related as reported for TiO_2_, ZnO, and hydroxides in the given references. The values given in the references labelled by * have been recalculated to a C1s binding energy fixed at 284.8 eV.

| Reference | TiO_2_ | ZnO | (Na)OH | H_2_O |
| --- | --- | --- | --- | --- |
| (Naseri, N. et al 2011) | ∗ 529.9 eV | 530.6 eV |  |  |
| (Zárate, R.A. et al 2007) | 529.8 eV | - | 533.3 eV |  |
| (Zárate, R.A. et al 2007) Mixture | ∗ 529.7 eV | - | 532.1 eV |  |
| (Du, P. et al 2012) | 526.8 eV | 527.1 eV |  |  |
| (Giannakopoulou, N. et al 2014) | ∗ 529.7 eV | 530.6 eV | 531.9 eV |  |
| (Wang N. et al 2008) | 529.6 eV | 531.1 eV |  |  |
| (Zhao, Y. et al 2008) | ∗ 529.3 eV-529.5 eV |  | 531.8 eV-532.0 eV |  |
| (Hugenschmidt et al 1994) | 530.2 eV |  |  | 532.9 eV |
| (Su et al 2013) |  | 530.0 eV | 531.5 eV | 533.1 eV |


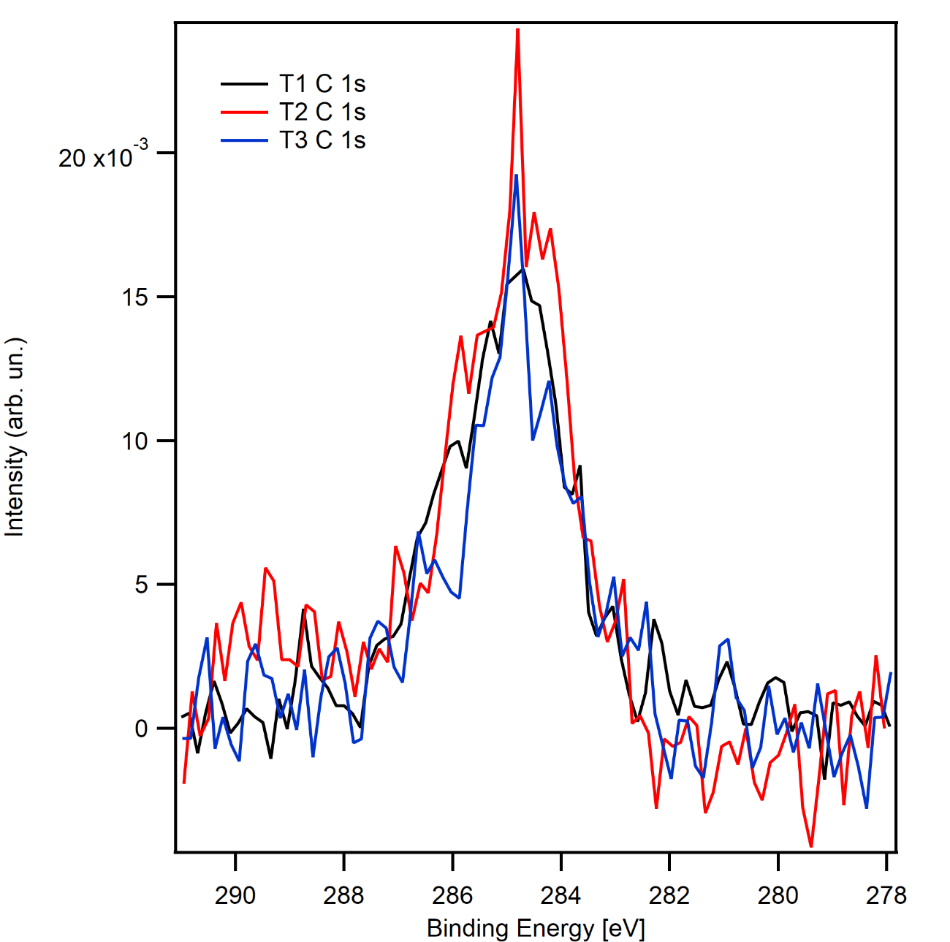


Figure S25. Normalized XPS spectra of the C1s region for samples T1-T3.


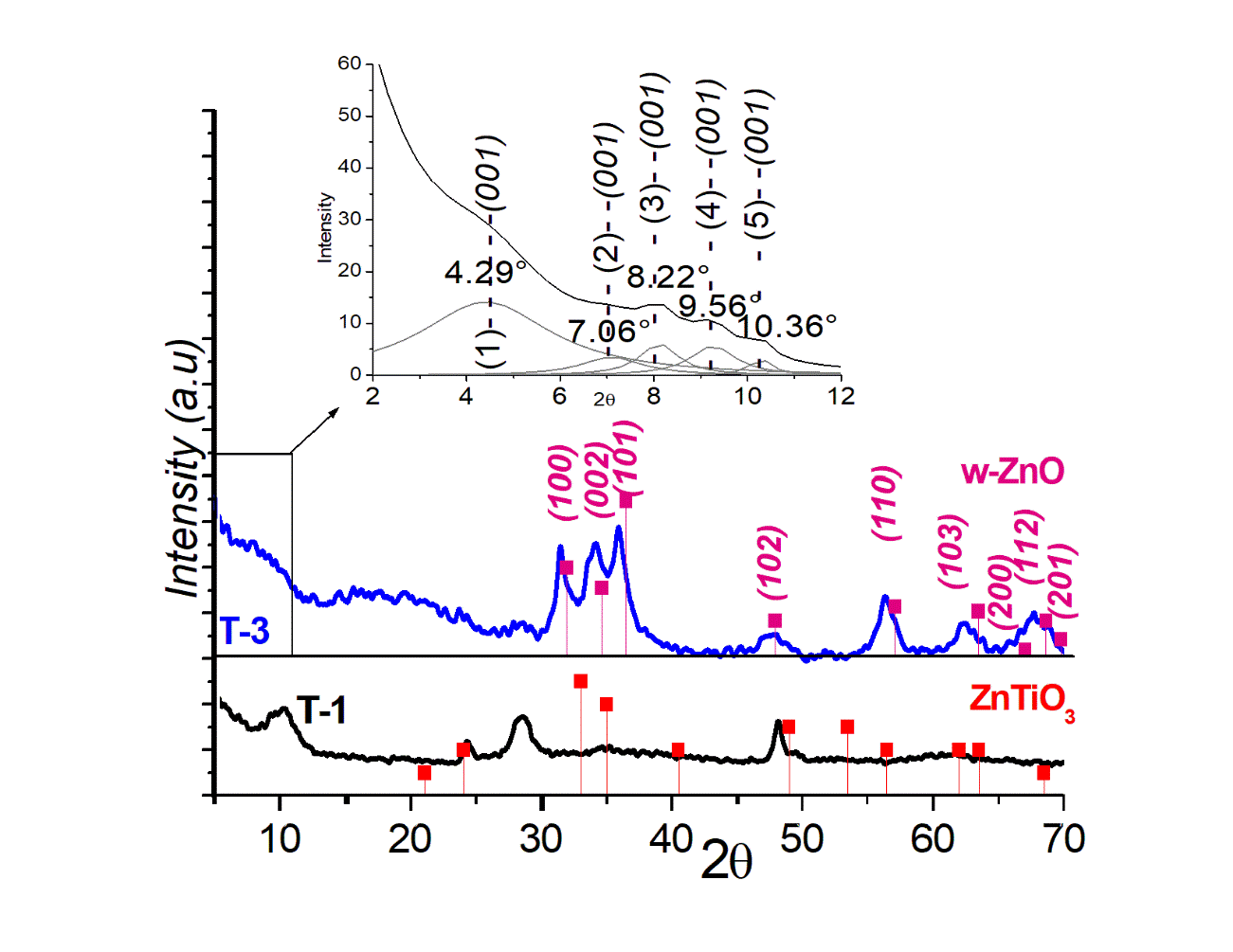


Figure S26. XRD of T3 (blue), w-ZnO (magenta) ICDD card 361451, ZnTiO_3_ (red) ICDD 26-150, and T1 (black), in range 2.5-5° in 2θ, showing *(00l)* family of planes (left-inset).


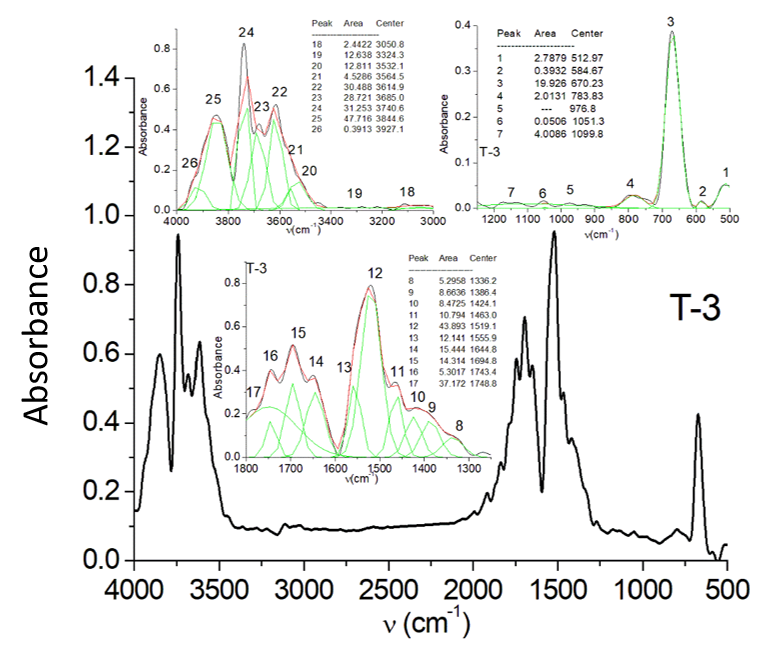


Figure S27. T3 IR spectrum in range of 500-4000 cm^-1^. Deconvoluted IR bands in the range of 500-1250 cm^-1^ (right inset), 3000-4000 cm^-1^(left inset) and 1000-1800 cm^-1^ (down-inset). In the IR spectrum of sample T3, the absorbance of the material is quite small. This is probably due to the nature of the observed absorptions, which are mostly surface vibration modes. The band prominence in the water spectral regions with respect to those involving oxygen–metal bonds observed in the spectrum would be due to differences in both concentration and oscillator strength of the infrared-sensitive functional groups existing in both moieties.


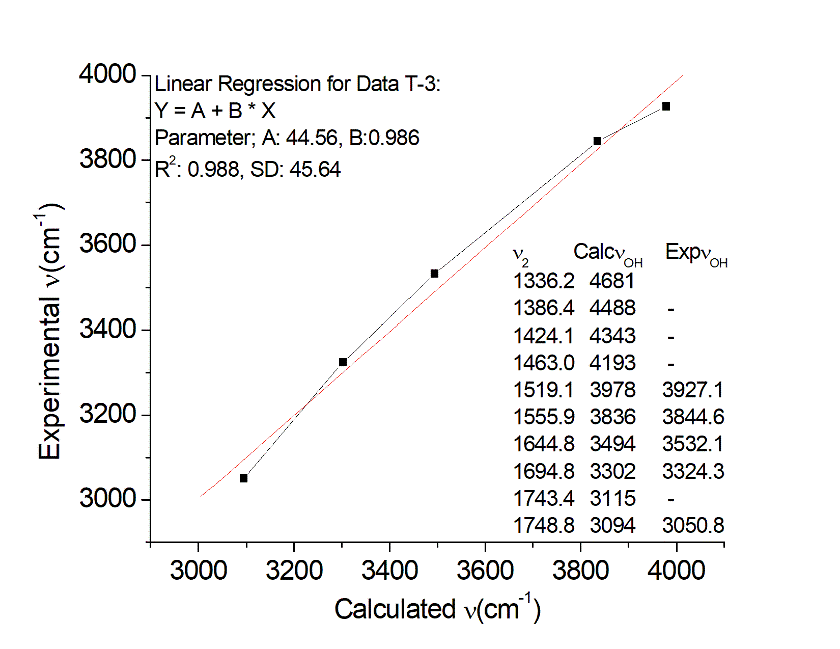


Figure S28. T3 IR linear correlation between calculated (Falk´s equation) and experimental νOH.

Table S5. Luminescence peaks of TiO_2_ anatase, w-ZnO, and T1, T2 and T3 in nm and eV.

| TiO_2_ anatase | T1 | T2 | T3 | w-ZnO nm (eV)  (Segovia M., et al. 2011) |
| --- | --- | --- | --- | --- |
| 365.10 ± 0.00 (3.40 ± 0.00) | 364.28 ± 1.25  (3.40 ± 0.01) | 363.03 ± 0.62  (3.41 ± 0.06) | 362.32 ± 0.09  (3.42±0.00) | 374 (3.31) |
| ------ | ----- | 389.38 ± 0.00  (3.18 ± 0.00) | 391.22 ± 0.01  (3.17 ± 0.00) | 385 (3.22) |
| 423.52 ± 0.90 (2.93 ± 0.01) | 424.33 ± 1.15  (2.92 ± 0.01) | 424.67 ± 0.24  (2.92 ± 0.00) | 424.74 ± 0.31  (2.92 ±0.00) | 423 (2.92) |
| 435.91 ± 0.16 (2.84 ± 0.00) | 436.44 ± 0.51  (2.84 ± 0.00) | N/O | 438.93 ± 0.01  (2.82 ± 0.00) | ------- |
| 449.09 ± 0.16 (2.76 ±.0.00) | 448.56 ±1.01  (2.76 ± 0.01) | 448.97 ± 0.63  (2.76 ± 0.00) | 448.08 ± 1.53  (2.76 ± 0.00) | -------- |
| 462.03 ± 0.05 (2.68 ± 0.00) | 462.73 ± 1.36  (2.68 ± 0.01) | 464.15 ± 0.76  (2.67 ± 0.00) | 463.32 ± 0.07  (2.67 ± 0.00) | ---------- |
| 487.96 ± 0.08 (2.54 ± 0.00) | 488.52 ± 1.02  (2.54 ± 0.01) | 489.70 ± 0.29  (2.53 ± 0.00) | 489.04 ± 0.79  (2.54 ± 0.00) | 490 (2.54) |
|  |  | 530.52 ± 6.32  (2.34±0.03) | 533.35 ± 9.15  (2.33±0.03) | 530(2.33) |

ND: not detected. O: overlapped.

1. **Photocatalytic properties of VPM and MPI TiO_2_/ZnO composites.**

To evaluate the semiconductor capacity of materials prepared using ALD-type techniques for operation in devices for the conversion of photonic energy into chemical energy, we analyze, as proof of concept, the catalytic activity of the samples prepared by VPM methods and MPI for degradation of organic pollutants. Specifically, we evaluate the photocatalytic activity of T2 and T3 samples in the degradation of methylene blue (MB) in water under UV illumination (300 W mercury lamp).

Typically, 2 x 10^-3^ g of the catalyst sample and 50 ml of 2 x 10^-5^ M aqueous solution of methylene blue were transferred to a photolysis reactor (López-Cabaña Z. et al., 2011). The suspension was left in the dark for at least 1 h to achieve adsorption balance. The temperature was maintained at 25 ° C throughout the process by a water-cooling jacket. Dye discoloration during irradiation was monitored by analysing the UV-visible absorption spectrum (Shimadzu UV-2450) of solution samples taken every 10 minutes under uninterrupted irradiation.

Figure S29 shows the photocatalytic degradation of MB assisted by T2 and T3 as a function of irradiation time. The results confirm that both products show photocatalytic activity and that their efficiency depends largely on the nature of the ALD treatment. The photocatalytic efficiency of the T2 sample with the ZnO nanoparticles interspersed between the TiO_2_ nanotube wall layers was practically zero, while the photocatalytic activity of the T3 sample, consisting mainly of single-wall trititanate nanotubes decorated by ZnO nanoparticles relatively large and / or coated by a thin film of microcrystalline ZnO, it was noticeably better. We attribute the low photocatalytic efficiency of the tubes after VPM treatment to the segmented nature of the ZnO phase in this product. The formation of oxide particles in the interlaminar spaces of the nanotube walls would not only be inhibiting a beneficial mobility, characteristic for TiO_2_/ZnO heterojunctions (Cheng. C., et al 2014, Hernández, S., Cauda, et al., 2014, Rudd, A. L., et al., 2000), but apparently, they would also be acting as traps that promote photogenerated charge recombination. On the other hand, the very small thin layer of ZnO that covers the surface of the nanotubes would create a synergistic system that manages to reduce the recombination rate of photoinduced charges (Scheme S1 in ESI). Such an interpretation is consistent with the low intensity of emission band observed around 449 nm, which we suggest corresponds to TiO_2_. Despite their exploratory nature, these experiments allow us to conclude that the intrinsic semiconducting properties of the oxides of both host and guest metal involved in the system are maintained after the ALD-type treatments, and that the combination of both VPM and MPI provides much room for developing composed semiconductors with regulable photocatalytic properties.


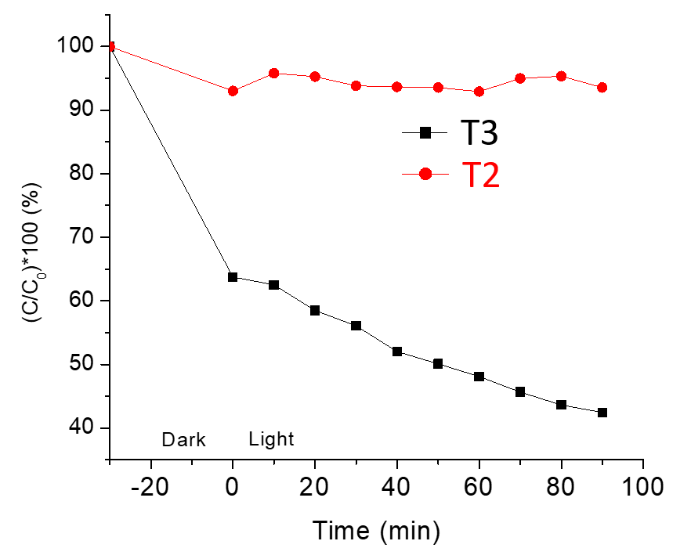


Figure S29. Photodegradation of methylene blue using T2 and T3.

1. **References**

Bradley M. (2007). Curve fitting in Raman and IR spectroscopy: basic theory of line shapes and applications. *Thermo Fisher Scientific, Madison, USA, Application Note.*, 50733.

Bunn C. W. (1935). The lattice-dimensions of zinc oxide. *Proceedings of the Physical Society., 47*(5), 835.

Caretti, I. Y. (2012). Coordination chemistry of titanium and zinc in Ti_(1− x)_Zn_2x_ O_2_ (0≤ x≤ 1) ultrathin films grown by DC reactive magnetron sputtering. *RSC Advances.,* , 2696-2699.

Cheng X, L. F. (2009). A DFT investigation on ZnO clusters and nanostructures. *Journal of Molecular Structure: THEOCHEM.* , 121-7.

Cheng, C., Amini, A., Zhu, C., Xu, Z., Song, H., & Wang, N. (2014). Enhanced photocatalytic performance of TiO_2_-ZnO hybrid nanostructures. *Scientific reports*, *4*, 4181.

Devlin JP, S. J. (2011). Infrared spectra of large H_2_O clusters: New understanding of the elusive bending mode of ice. . *The Journal of Physical Chemistry A.* , 974-83.

Du, P. et al 2012. Coaxial Electrospun TiO2/ZnO Core–Sheath Nanofbers Film: Novel Structure for Photoanode of Dye-Sensitized Solar Cells. Electrochimica Acta 78, pp. 392–397. issn: 00134686. doi: 10.1016/j.electacta.2012.06.034.

Falk M. (1984). The frequency of the H-O-H bending fundamental in solids and liquids. *Spectrochimica Acta Part A: Molecular Spectroscopy.*, 43-48.

Feist TP, D. P. (1992). The soft chemical synthesis of TiO_2_ (B) from layered titanates. *Journal of solid state chemistry.*, 275-295.

Giannakopoulou T. et al (2014). Optical and Photocatalytic Properties of Composite TiO_2_/ZnO Thin Films. Catalysis Today 230, pp. 174–180. issn: 09205861. doi: 10.1016/j.cattod.2013.10.003.

Hernández, S., Cauda, V., Chiodoni, A., Dallorto, S., Sacco, A., Hidalgo, D., & Pirri, C. F. (2014). Optimization of 1D ZnO@TiO_2_ core–shell nanostructures for enhanced photoelectrochemical water splitting under solar light illumination. *ACS applied materials & interfaces*, *6*(15), 12153-12167.

Hugenschmidt, M. B., Gamble, L., & Campbell, C. T. (1994). The interaction of H2O with a TiO2 (110) surface. Surface Science, 302(3), 329-340.

Iordanova R, B.-N. A. (2011). Mechanochemical synthesis and photocatalytic properties of zinc titanates. . *Bulgarian Chemical Communications.*, 378-382.

Jackson P, P. G. (1971). Infra-red study of the surface properties of rutile. Water and surface hydroxyl species. . *Transactions of the Faraday Society.* , 2469-83.

Kevorkyants R, R. A. (2016). The origin of 1560 cm^−1^ band in experimental IR spectra of water adsorbed on TiO_2_ surface: Ab initio assessment. . *Chemical Physics Letters.* 97-101.

Lavrov EV, W. J. (2002). Hydrogen-related defects in ZnO studied by infrared absorption spectroscopy. *Physical Review B.* 165205.

López-Cabaña, Z. T. C. M. S. &. G. G. (2011). Semiconducting properties of layered cadmium sulphide-based hybrid nanocomposites. Nanoscale research letters, 6((1), 523.

Max JJ, C. C. (2009). Isotope effects in liquid water by infrared spectroscopy. III. H_2_O and D_2_O spectra from 6000 to 0 cm^− 1^. *The Journal of chemical physics.* 184505.

Max JJ, C. C. (2011). Isotope effects in liquid water by infrared spectroscopy. V. A sea of OH_4_ of C_2v_ symmetry. *The Journal of chemical physics.*, 164502.

Ni Y, S. J. (2015). IR and SFG vibrational spectroscopy of the water bend in the bulk liquid and at the liquid-vapor interface, respectively. . *The Journal of chemical physics.* , 014502.

Naseri N. et al 2011. A Comparative Study on Photoelectrochemical Activity of ZnO/TiO_2_ and TiO_2_/ZnO Nanolayer Systems under Visible Irradiation. Solar Energy 85.9, pp. 1972–1978. doi: 10.1016/j.solener. 2011.05.002.

Ogasawara, H. et al 2008. Double role of water in the fuel cell oxygen reduction reaction. *ECS Transactions*, *16*(2), 1385.

Pingfan Du et al 2012. Coaxial Electrospun TiO_2_/ZnO Core–Sheath Nanofbers Film: Novel Structure for Photoanode of Dye-Sensitized Solar Cells. Electrochimica Acta 78, pp. 392–397. doi: 10.1016/j.electacta.2012.06.034.

Pejov L, J. G. (2017). Low bending vibrations of crystalline water molecules: An ongoing quest or a final word-topical review-a tribute to Academican Bojan Soptrajanov. *Contributions, Section of Natural, Mathematical and Biotechnical Sciences.* 69-82.

Petruševski V, Š. B. (1990). Vibrational spectra of hexaaqua complexes: I. Assignments of water librational bands in the spectra of some alums. *Journal of Molecular Structure.* 67-72.

Qamar M, Y. C. (2008). Preparation and photocatalytic activity of nanotubes obtained from titanium dioxide. . *Catalysis Today*. 3-14.

Ruiz-Fuertes J, W. B.-M. (2015). Ferroelectric soft mode of polar ZnTiO_3_ investigated by Raman spectroscopy at high pressure. *Physical Review B.* 214110.

Rudd, A. L., & Breslin, C. B. (2000). Photo-induced dissolution of zinc in alkaline solutions. *Electrochimica acta*, *45*(10), 1571-1579.

Soria J, S. J.-A. (2007). FTIR and NMR study of the adsorbed water on nanocrystalline anatase. *The Journal of Physical Chemistry C.* 10590-10596.

Sumita M, H. C. (2010). Interface water on TiO_2_ anatase (101) and (001) surfaces: First-principles study with TiO_2_ slabs dipped in bulk water.*The Journal of Physical Chemistry C.*, 18529-18537.

Su, C. Y., Hsueh, Y. C., Kei, C. C., Lin, C. T., & Perng, T. P. (2013). Fabrication of high-activity hybrid Pt@ ZnO catalyst on carbon cloth by atomic layer deposition for photoassisted electro-oxidation of methanol. *The Journal of Physical Chemistry C*, *117*(22), 11610-11618.

Vinaykin M, B. A. (2012). Vibrational sum-frequency spectrum of the water bend at the air/water interface. *The Journal of Physical Chemistry Letters.* 3348-3352.

Wagner C. D. et al (1979). Handbook of X-Ray Photoelectron Spectroscopy. Minnesota: Perkin-Elmer, Co.

Wang X, A. L. (2005). Infrared spectra and structures for group 4 dihydroxide and tetrahydroxide molecules. *The Journal of Physical Chemistry A.* 10689-10701.

Wang N. et al (2008). Synthesis of ZnO/TiO_2_ Nanotube Composite Film by a Two-Step Route. Materials Letters 62.21-22, pp. 3691–3693. issn: 0167577X. doi: 10.1016/j.matlet.2008.04.052.

Yamaguchi O, M. M. (1987). Formation and transformation of ZnTiO_3_. *J. Am. Ceram. Soc.* 5.

Yates DJ. (1961). Infrared studies of the surface hydroxyl groups on titanium dioxide, and of the chemisorption of carbon monoxide and carbon dioxide. *J. Phys. Chem.* 746-53.

Yin Zhao et al (2008). Zn-Doped TiO_2_ Nanoparticles with High Photocatalytic Activity Synthesized by Hydrogen–Oxygen Difusion Flame. Applied Catalysis B: Environmental 79.3, pp. 208–215. issn: 09263373. doi: 10.1016/j.apcatb.2007.09.044.

Zarate R.A. et al (2007). Chemical Composition and Phase Identifcation of Sodium Titanate Nanostructures Grown from Titania by Hydrothermal Processing. Journal of Physics and Chemistry of Solids 68.4, pp. 628–637. issn: 00223697. doi: 10.1016/j.jpcs.2007.02.011.
